# Two nuclear effectors of the rice blast fungus modulate host immunity via transcriptional reprogramming

**DOI:** 10.1038/s41467-020-19624-w

**Published:** 2020-11-17

**Authors:** Seongbeom Kim, Chi-Yeol Kim, Sook-Young Park, Ki-Tae Kim, Jongbum Jeon, Hyunjung Chung, Gobong Choi, Seomun Kwon, Jaeyoung Choi, Junhyun Jeon, Jong-Seong Jeon, Chang Hyun Khang, Seogchan Kang, Yong-Hwan Lee

**Affiliations:** 1 Department of Agricultural Biotechnology, Seoul National University, Seoul, 08826 Korea; 2 Department of Plant Medicine, Sunchon National University, Suncheon, 57922 Korea; 3 Interdisciplinary Program in Agricultural Genomics, Seoul National University, Seoul, 08826 Korea; 4 Department of Biotechnology, Yeungnam University, Gyeongsan, 38541 Korea; 5 Graduate School of Biotechnology and Crop Biotech Institute, Kyung Hee University, Yongin, 17104 Korea; 6 Department of Plant Biology, University of Georgia, Athens, GA 30602 USA; 7 Department of Plant Pathology and Environmental Microbiology, The Pennsylvania State University, University Park, PA 16802 USA; 8 Center for Fungal Genetic Resources, Plant Immunity Research Center, and Research Institute of Agriculture and Life Sciences, Seoul National University, Seoul, 08826 Korea; 9 Present Address: Crop Cultivation and Environment Research Division, National Institute of Crop Science, Rural Development Administration, Suwon, 16613 Korea; 10 Present Address: Heinrich-Heine University Düsseldorf, Institute for Microbiology, Cluster of Excellence on Plant Sciences, Düsseldorf, 40204 Germany; 11 Present Address: Smart Farm Research Center, Korea Institute of Science and Technology, Gangneung, 25451 Korea

**Keywords:** Agricultural genetics, Fungal pathogenesis, Effectors in plant pathology

## Abstract

Pathogens utilize multiple types of effectors to modulate plant immunity. Although many apoplastic and cytoplasmic effectors have been reported, nuclear effectors have not been well characterized in fungal pathogens. Here, we characterize two nuclear effectors of the rice blast pathogen *Magnaporthe oryzae*. Both nuclear effectors are secreted via the biotrophic interfacial complex, translocated into the nuclei of initially penetrated and surrounding cells, and reprogram the expression of immunity-associated genes by binding on effector binding elements in rice. Their expression in transgenic rice causes ambivalent immunity: increased susceptibility to *M*. *oryzae* and *Xanthomonas oryzae* pv. *oryzae*, hemibiotrophic pathogens, but enhanced resistance to *Cochliobolus miyabeanus*, a necrotrophic pathogen. Our findings help remedy a significant knowledge deficiency in the mechanism of *M*. *oryzae*–rice interactions and underscore how effector-mediated manipulation of plant immunity by one pathogen may also affect the disease severity by other pathogens.

## Abstract (web summary)

Plant pathogens secrete various effectors to manipulate host immunity. Here, Kim et al. describe two *Magnaporthe oryzae* effectors that translocate into the nuclei of infected rice cells and reprogram expression of immunity-associated genes, increasing susceptibility to hemibiotrophic pathogens.

## Introduction

Plants employ two-layered immune systems for protection from diverse groups of pathogens. Cell surface pattern recognition receptors (PRRs) detect conserved pathogen-associated molecular patterns (PAMPs) to trigger PAMP-triggered immunity (PTI)^1^. Effector-triggered immunity (ETI) gets activated when specific host resistance proteins recognize matching pathogen effectors directly or indirectly. Generally, while PTI confers basal and broad-spectrum resistance, ETI provides race-specific resistance with stronger defense responses, such as the hypersensitive response^1^. Both types of immunity entail large-scale transcriptional reprogramming orchestrated by phytohormones and transcription factors^2,3^.

Pathogens elude or suppress host defense by releasing effectors that collectively function to manipulate pathogen surveillance systems, physiology, and immune responses. Effectors often target host receptors or defense-signaling components to disrupt the initiation of defense responses^4^. Pathogen effectors typically display a high level of genetic diversity due to arms races between plants and pathogens and can be classified according to where they function. Apoplastic effectors are secreted to the outside of plant cells, while cytoplasmic effectors are translocated into the plant cytoplasm via several mechanisms. Several effectors that function in plant nuclei have been identified in oomycetes and bacterial pathogens. *Phytophthora* Crinkler (CRN) effectors and bacterial Transcription Activator-Like (TAL) effectors suppress host defense or induce susceptibility by manipulating plant gene expression^5,6^. Compared to research on such bacterial and oomycete effectors, research on fungal nuclear effectors lags. Nuclear effector candidates in fungal pathogens, including *Ustilago maydis* See1, *Puccinia striiformis* f.sp. *tritici* PstGSRE1, and *Colletotrichum graminicola* CgEP1, have been reported^7–9^. However, more work is needed to confirm nuclear localization during infection or whether they control the expression of immunity-associated genes in hosts.

Rice blast, caused by *M*. *oryzae*, reduces global rice yield by 10–30% annually^10^. Besides its economic significance, this pathosystem has been extensively investigated, resulting in high-quality genome and transcriptome data for rice and *M. oryzae*. Additionally, protocols for transformation and gene manipulation of both organisms are robust. *M*. *oryzae* forms an infection structure called the appressorium to penetrate host cells and develops invasive hyphae (IH) in penetrated cells without causing cell death^11^. Cytoplasmic effectors are secreted into the cytoplasm of invaded rice cells via the biotrophic interfacial complex (BIC)^11^. Its apoplastic effectors are secreted into the extra-invasive hyphal membrane (EIHM) compartment^11^.

In this study, we identify and characterize two nuclear effectors of *Magnaporthe oryzae*, named as MoHTR1 and MoHTR2 for *M*. *oryzae* Host Transcription Reprogramming 1 and 2, respectively. Both MoHTR1 and MoHTR2 seem to function as transcription repressors that reprogram the transcription of large numbers of immunity-associated genes in rice. We also identify effector binding elements and uncover candidate target genes of these nuclear effectors. Interestingly, transgenic expression of *MoHTR1* and *MoHTR2* in rice affects susceptibility to not only *M. oryzae* but also *Xanthomonas oryzae* pv. *oryzae*, a hemibiotrophic bacterial pathogen, and *Cochliobolus miyabeanus*, a necrotrophic fungal pathogen, in a manner that depends on their mode of infection. Our findings suggest that these nuclear effectors modulate disease susceptibility of rice via transcriptional reprogramming of immunity-associated genes.

## Results

### Identification of nuclear effectors of *M*. *oryzae*

We identified nuclear effectors based on the presence of both a secretion signal and a DNA-binding domain. In total, 1895 *M*. *oryzae* proteins that lack a glycosylphosphatidylinositol (GPI) anchor, an endoplasmic reticulum (ER) retention signal, and transmembrane helix(es) were curated as secreted proteins in Fungal Secretome Database (FSD; http://fsd.snu.ac.kr)^12^. Among these, nuclear effector candidates that likely participate in transcriptional regulation were identified using Fungal Transcription Factor Database (FTFD; http://ftfd.snu.ac.kr)^13^. Twenty proteins carry a DNA-binding domain, with 50% of them possessing the C_2_H_2_ zinc finger domain (IPR007087). The remaining proteins carry one of the following domains: high mobility group superfamily (IPR009071), Myb (IPR001005), homeodomain-like (IPR009057), Zn_2_Cys_6_ transcription factor (IPR007219), TFIIS zinc finger (IPR012164), and nucleic acid-binding OB fold (IPR008994). They were named as MoHTR1-20 for *M. oryzae* Host Transcription Reprogramming 1–20 (Supplementary Table 1). We used WoLF-PSORT^14^, NLStradamus^15^, and cNLS mapper^16^ to check the presence of a potential nuclear localization signal (NLS) in MoHTR1-20. Sixteen proteins were predicted to carry one or more NLS by at least one program (Supplementary Table 1).

### Expression patterns and homolog distribution and structural features of MoHTRs

We analyzed how the MoHTR genes are expressed during vegetative growth and in infected rice sheaths at the pre-penetration (18 hours post inoculation, hpi), biotrophic (27 and 36 hpi), transition (45 hpi), and necrotrophic (72 hpi) stages using available RNA-seq data^17^. Eleven genes were highly expressed during the biotrophic or transition stage, with their log_2_(FPKM+1) being ≥ 4. Among these, eight genes were considered infection-specific because their transcripts were undetectable or very low in vegetative mycelia (Supplementary Fig. 1).

We used BLASTMatrix, a BLAST tool designed to search multiple genomes simultaneously, in Comparative Fungal Genomics Platform 2.0 (CFGP 2.0)^18^ to search their homologs in diverse species. Genes homologous to some MoHTR genes were present in 97 fungi, 5 oomycetes, 2 plants, and 3 animals. Homologs of *MoHTR11*, *MoHTR13*, *MoHTR14*, *MoHTR15*, and *MoHTR19* are present in most fungal and oomycete species analyzed. Homologs of *MoHTR12*, *MoHTR16*, *MoHTR18*, and *MoHTR20* are present in subclades of Ascomycota and Basidiomycota but mostly absent among oomycetes. The remaining 11 genes appear *M*. *oryzae*-specific. Distribution patterns of all MoHTR genes at the population level were also analyzed using the genomes of 39 *M*. *oryzae* isolates. Fifteen genes were present in more than 36 isolates.

Two *M. oryzae*-specific effectors, MoHTR1 and MoHTR2, were selected for further analyses because they were specifically expressed during infection (Supplementary Fig. 1) and accumulated at BIC. We compared their structural features with *Drosophila melanogaster* GAGA, a well-characterized C_2_H_2_ zinc-finger transcription factor, to infer how MoHTR1 and MoHTR2 interact with DNA. The binding of GAGA to target gene promoters requires a single C_2_H_2_ zinc finger domain and flanking basic amino acid-rich regions^19^. The structure of the C_2_H_2_ zinc finger domain in MoHTR1 and MoHTR2 was predicted via ab initio modeling (Phyre2; http://www.sbg.bio.ic.ac.uk/~phyre2)^20^. The corresponding domain of *D. melanogaster* GAGA was superimposed to those of MoHTR1 and MoHTR2 using SuperPose version 1.0 (http://wishart.biology.ualberta.ca/SuperPose/)^21^. The root mean square deviation (RMSD) between MoHTR1 and GAGA was 2.41 Å and that between GAGA and MoHTR2 was 1.95 Å. Similar to GAGA, basic amino acids are present at both sides of the zinc finger domain of MoHTR1 and MoHTR2, suggesting their DNA binding.

### In planta localization of MoHTR1 and MoHTR2

**Fig. 1:**
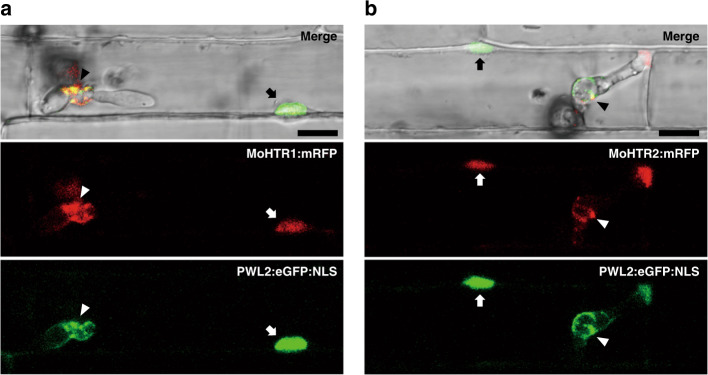
Localization of MoHTR1 and MoHTR2 in rice nuclei. Differential interference contrast (DIC) and confocal images of rice sheath cells infected by *M*. *oryzae* at 30 hours post-inoculation (hpi). The fluorescent images shown were chosen from confocal optical z-sections taken at 1-μm intervals. Both **a** MoHTR1:mRFP and **b** MoHTR2:mRFP were co-localized with PWL2:eGFP:NLS, a maker used to label rice nuclei (arrow). Arrowheads indicate biotrophic interfacial complexes of invading *M*. *oryzae*. Scale bars = 10 μm. Representative micrographs are shown from three independently infected rice sheaths and additional representative data are provided as a Source Data file.

To determine where MoHTR1 and MoHTR2 localize during rice infection, fungal transformants expressing these proteins fused to a monomeric RFP (MoHTR:mRFP) under their native promoters were generated. Rice sheaths inoculated with individual transformants were imaged at 30 hpi. Consistent with their transcriptional induction during the biotrophic stage, both MoHTR1:mRFP and MoHTR2:mRFP accumulated in BICs and rice nuclei (Fig. 1). Rice nuclei were marked by expressing PWL2, a known cytoplasmic effector of *M*. *oryzae*^11^, fused to eGFP:NLS (eGFP linked to a NLS of SV40 large T antigen). MoHTR1:mRFP and MoHTR2:mRFP were detected in rice nuclei in 77% (*n* = 43) and 89% (*n* = 37) of the infection sites observed, respectively. When fungal hyphae moved into the cells adjacent to initially penetrated cells, both proteins were also detected in their nuclei (Supplementary Fig. 2).

**Fig. 2:**
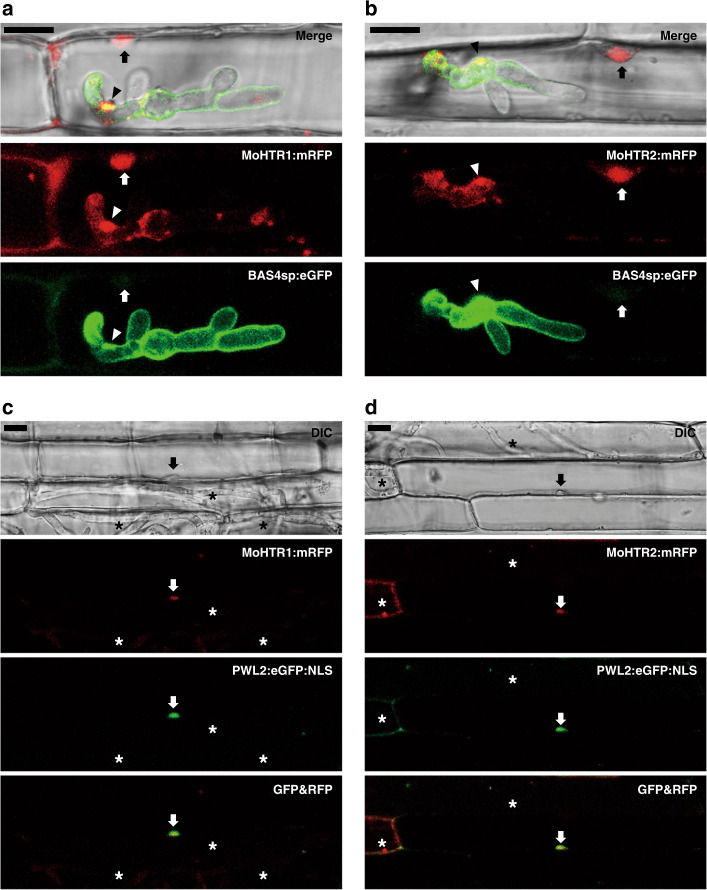
Translocation of MoHTR1 and MoHTR2 into rice cell and migration into rice nuclei. The MoHTR1:mRFP and MoHTR2:mRFP expressed under control of their native promoter were observed at an early infection stage (30 hpi). BAS4sp:eGFP is a marker for intact extra-invasive hyphal membrane. **a** MoHTR1:mRFP and **b** MoHTR2:mRFP were mainly localized in BICs and the nuclei of invaded rice leaf sheath cells. The images shown were chosen from confocal optical z-sections taken at 1-μm intervals. At 48 hpi, **c** MoHTR1:mRFP and **d** MoHTR2:mRFP were also detected in the nuclei of some uninfected cells. Arrowheads indicate BICs, and arrows indicate rice nuclei. Asterisks indicate rice cells invaded by fungal hyphae. Scale bars = 10 μm. Representative micrographs are shown from three independently infected rice sheaths and additional representative data are provided as a Source Data file.

Penetration of *M*. *oryzae* results in the invagination of the rice plasma membrane. Because the rice plasma membrane remains intact during the biotrophic stage of infection^22^, pathogen effectors must infiltrate the membrane to translocate into rice cells. BAS4 is an apoplastic effector of *M*. *oryzae*, and its secretion signal peptide (BAS4sp) linked to a fluorescent protein has been used as a marker for checking the integrity of the rice membrane^11^. When rice sheaths were inoculated with fungal transformants expressing both BAS4sp:eGFP and each MoHTR:mRFP, BAS4sp:eGFP was retained in the apoplast whereas MoHTR1:mRFP and MoHTR2:mRFP were detected in rice nuclei (Fig. 2a, b). *M*. *oryzae* cytoplasmic effectors have been shown to move to as-yet-uninvaded rice cells through the plasmodesmata^11^. Similarly, MoHTR1:mRFP and MoHTR2:mRFP were also detected in nuclei of uninvaded rice cells (Fig. 2c, d). The MoHTR1 and MoHTR2 genes that lack the secretion signal peptide (MoHTR1-Δsp and MoHTR2-Δsp) were fused to *eGFP* to check their subcellular location in rice protoplasts. Consistent with their nuclear localization in infected rice sheath cells, MoHTR1-Δsp and MoHTR2-Δsp were mostly located in rice nuclei (Supplementary Fig. 3). Rice nuclei were marked using ABF1, a rice transcription factor^23^. We also determined whether both MoHTR-Δsp proteins localize in fungal nuclei. MoHTR1-Δsp:mRFP was detected in the cytoplasm, and MoHTR2-Δsp:mRFP appeared as speckles and did not accumulate in fungal nuclei (Supplementary Fig. 3).

### Identification of effector binding elements for MoHTR1 and MoHTR2

**Fig. 3:**
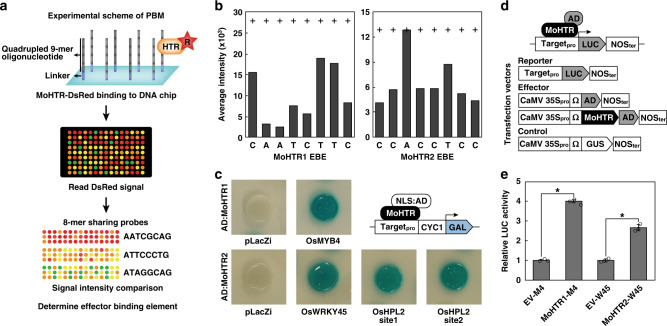
Identification of effector binding elements targeted by MoHTR1 and MoHTR2. **a** Effector binding elements (EBEs) targeted by MoHTR1 and MoHTR2 were identified using a protein binding microarray. Each MoHTR-Δsp:DsRed (R) protein produced using *E*. *coli* was applied to DNA chip containing quadrupled 9-mer nucleotides. The EBE for each protein was identified based on the fluorescent intensity from the group of elements containing the same 8-mer nucleotide. **b** Effect of a single nucleotide mismatch in each EBE on the binding affinity to MoHTR. The line denoted using + indicates the signal intensity from EBE without variation. Bars represent the average signal intensity from individual EBE variants with each carrying a different single-nucleotide mismatch (noted in *x*-axis). **c** Schematic representation of a yeast one-hybrid assay used for detecting MoHTR–EBE interaction and resulting data. NLS:AD:MoHTR-Δsp was expressed under the ADH1 promoter. NLS, a nuclear localization signal from the Simian Virus 40 large T antigen; AD, GAL4 activation domain. The reporter, β-galactosidase (GAL), was placed under the recombinant promoter comprised of an EBE-centered 38-bp fragment and a minimal cytochrome C1 promoter (CYC1). GAL expression was activated when NLS:AD:MoHTR-Δsp binds to its EBE in the promoter. pLacZi was used as a negative control. **d** Validation for binding of MoHTRs and target promoters in planta. *LUC* was regulated by the corresponding gene promoter. Activation domain of TAL effector (AD) was linked to *MoHTR*-Δsp and expressed under the 35S CaMV promoter. **e** Relative LUC activities in rice protoplasts transfected with AD-linked *MoHTR*-Δsp were compared with those transfected with the EV and corresponding reporter vector (M4: *OsMYB4* promoter, W45: *OsWRKY45* promoter). *n* = 3 independently transfected protoplasts; mean ± standard deviation (SD); *P < 0.01, two-sided Student’s *t*-tests. Source data underlying Fig. 3e are provided as a Source Data file.

The presence of a C_2_H_2_ zinc finger domain in MoHTR1 and MoHTR2 and their accumulation in rice nuclei led to the hypothesis that they regulate the transcription of rice genes by binding to target gene promoters. We identified candidate binding sequences using a quadruple 9-mer-based protein binding microarray (PBM)^24^ (Fig. 3a). This approach allowed us to quantify the binding affinity of DsRed-linked MoHTR1 and MoHTR2 to all possible 9-mer DNA sequence probes based on the fluorescence intensity from individual probes on the chip. The rank-ordered signal distribution exhibited a steep rightward slope, indicating that these proteins strongly bind to only a small number of DNA probes. Each 8-mer sequence occurs 36 times in this 9-mer-format DNA microarray. We sorted the median signal intensity for all possible 8-mers to identify effector binding elements (EBEs) (Supplementary Table 2). The highest median intensity was found at CAATCTTC for MoHTR1 and CCACCTCC for MoHTR2. We compared the average signal intensities from EBE variants with a single-nucleotide variation to determine which nucleotide(s) are crucial for binding. All nucleotide changes in the MoHTR1 EBE reduced the signal intensity at varying degrees. The second and third nucleotides appeared most important for MoHTR1 binding. For MoHTR2, all changes except the third position decreased the signal intensity (Fig. 3b).

### Identification of the rice genes targeted by MoHTR1 and MoHTR2

We mined the rice genome to identify the candidate genes targeted by MoHTR1 and MoHTR2 based on the presence of EBE in their promoter region (defined as 1-kb upstream sequences from the coding region) and differential expression during *M. oryzae* infection. We identified 226 (116 up- and 110 down-regulated) and 650 (274 up- and 376 down-regulated) genes that met both criteria as potential targets for MoHTR1 and MoHTR2, respectively (Supplementary Tables 3 and 4). Eleven genes appeared to be common targets of both effectors (Supplementary Table 5). Gene ontology (GO) enrichment analysis was performed using RiceNetDB^25^ to classify their functions. Metabolism-related and stress-responsive terms are commonly enriched among the candidates targeted by MoHTR1 and MoHTR2 (Supplementary Tables 6 and 7). Forty and 75 potential targets for MoHTR1 and MoHTR2, respectively, were associated with the term “Response to stress (GO: 0006950)”, which also includes disease resistance genes (Supplementary Tables 6 and 7). We also found previously characterized immunity-associated genes, including *OsMYB4* for MoHTR1 and *OsHPL2* and *OsWRKY45* for MoHTR2^26–28^. We determined whether MoHTR1 and MoHTR2 bind to their target gene promoters in vivo via yeast one-hybrid analysis. Both MoHTR-Δsps fused to the GAL4 activation domain (AD) were expressed using the yeast *ADH1* promoter. The 38-bp promoter fragments from three target genes, which included EBE, were used. Both AD:MoHTR1-Δsp and AD:MoHTR2-Δsp activated the reporter gene expression (Fig. 3c), suggesting that MoHTR1 and MoHTR2 bind to these promoters. No reporter gene expression was detected in the negative control.

We also performed a luciferase (LUC)-based assay by transiently expressing these MoHTR effectors in rice protoplasts to confirm their binding to selected target gene promoters in planta. The LUC reporter gene was cloned under the promoter of *OsMYB4* or *OsWRKY45*. The luminescence signal increased only when each MoHTR effector linked to the TAL activation domain was expressed in rice protoplasts (Fig. 3e), supporting that the MoHTR effectors interact with the target gene promoters.

### MoHTR1 and MoHTR2 regulate transcription in rice protoplasts

**Fig. 4:**
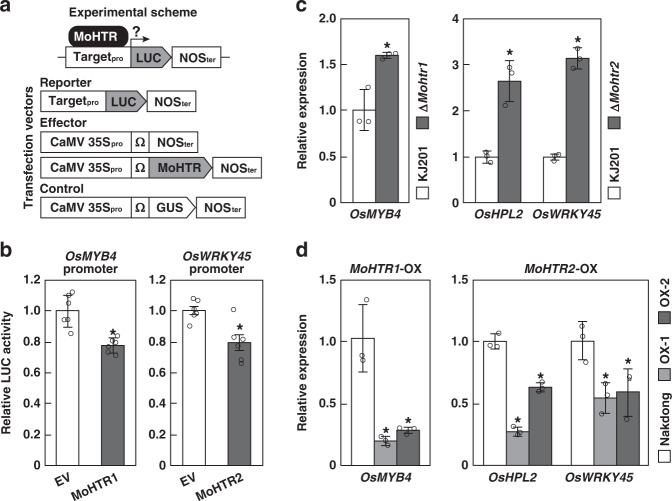
Transcriptional regulation of rice genes targeted by MoHTR1 and MoHTR2. **a** Features of the constructs used to determine how *MoHTR1* and *MoHTR2* affect the expression of LUC under each rice gene promoter tested in rice protoplasts. *MoHTR1*-Δsp and *MoHTR2*-Δsp were expressed using the CaMV 35S promoter and the translation enhance sequence Ω. **b** Relative LUC activities in protoplasts transfected with *MoHTR1*-Δsp and *MoHTR2*-Δsp were compared with those transfected with the empty vector (EV). *n* = 6 independently transfected protoplasts; mean ± SD; *P < 0.01, two-sided Student’s t-tests. Three biological replicates produced similar results. **c** Expression patterns of the selected target genes in rice individually infected with Δ*MoHTR1* and Δ*MoHTR2* were compared with those in rice infected with KJ201. Mean ± SD, three independent experiments; *P < 0.01, two-sided Student’s *t*-tests. **d** Expression levels of individual target genes in *MoHTR1*-OX and *MoHTR2*-OX were compared with those in Nakdong (wild type) using qRT-PCR. Mean ± SD, three independent experiments; *P < 0.05, one-way analysis of variance (ANOVA) with Tukey’s honest significant difference (HSD) test. Detailed information about biological repeat experiments and statistical analysis underlying Fig. 4b–d are described as a Source Data file.

We transiently expressed *MoHTR1* and *MoHTR2* in rice protoplasts to determine how they regulate rice gene expression. We designed to express the LUC gene under the control of target promoters of MoHTR effectors (*OsMYB4* for MoHTR1 and *OsWRKY45* for MoHTR2) to investigate the nature of transcriptional reprogramming by these effectors (Fig. 4a). Each MoHTR-Δsp suppressed the *LUC* expression (Fig. 4b), suggesting that both MoHTR1 and MoHTR2 function as a transcriptional repressor. We used the LUC gene under the control of GAL4 upstream activation sequence (UAS)-linked 35S minimal promoter (35S_mini_ promoter) to induce basal level expression. We also expressed GAL4 DNA binding domain (GBD)-linked MoHTR-Δsp for binding to UAS:35S_mini_ promoter (Supplementary Fig. 4). The basal expression of *LUC* under the UAS:35S_mini_ promoter was not changed by GBD:MoHTR-Δsp expression (Supplementary Fig. 4), suggesting that MoHTR1 and MoHTR2 may not have an intrinsic repressor activity and recruit unknown repressors(s).

### MoHTR1 and MoHTR2 reprogram the expression of many immunity-associated genes in rice

To investigate how MoHTR1 and MoHTR2 modulate the expression of target genes during infection, we generated deletion mutants of *MoHTR1* and *MoHTR2* in *M*. *oryzae* KJ201. We compared transcript levels of target genes in Δ*Mohtr*-infected rice plants with those in control plants (infected with KJ201). The transcript level of *OsMYB4* in Δ*Mohtr1*-infected rice increased 1.6-fold over that of the control. In Δ*Mohtr2*-infected plants, the expression of *OsHPL2* and *OsWRKY45* was induced by 2.6- and 3.1-folds, respectively (Fig. 4c). These results are consistent with the model that *MoHTR1* and *MoHTR2* encode transcription repressors for regulating expression of their target genes in rice.

We also generated transgenic lines of the rice cultivar Nakdong that express the *MoHTR1-*Δsp and *MoHTR2*-Δsp individually under the control of the CaMV 35S promoter. Transgenic lines that highly expressed *MoHTR1* (*MoHTR1*-OX) and *MoHTR2* (*MoHTR2*-OX) were chosen. Transcript levels of *MoHTR1* and *MoHTR2* in these *MoHTR*-OX lines were up to 6.5- and 9.6-folds higher than that of rice *Actin*. We analyzed how target genes in two independent *MoHTR*-OX lines for each effector were affected compared to their expression in Nakdong. The expression of *OsMYB4* decreased in the *MoHTR1*-OX lines (up to 0.2-fold). In the *MoHTR2*-OX lines, both *OsHPL2* and *OsWRKY45* were downregulated (up to 0.3- and 0.6-folds, respectively) (Fig. 4d). These results provide further evidence supporting the role of MoHTR1 and MoHTR2 as transcription repressors in rice.

MoHTR1 and MoHTR2 also affected the expression of other immunity-associated genes that were not directly targeted by these effectors. Expression of several immunity-associated genes such as pathogenesis-related (*PR*) and hormone signaling genes increased when rice was infected with Δ*Mohtr1* and Δ*Mohtr2* (Supplementary Fig. 5). Moreover, the expression of *PR5*, *PR10a*, and salicylic acid-related genes was downregulated in the *MoHTR*-OX lines (Supplementary Fig. 5). To identify rice genes affected by these nuclear effectors genome-wide, we performed RNA-seq analyses of *MoHTR1*-OX and *MoHTR2*-OX lines. More than 1,000 differentially expressed genes (DEGs) were found in both transgenic lines. A GO enrichment analysis revealed that the genes associated with “response to biotic stimulus (GO:0009607)” were commonly downregulated in *MoHTR1*-OX and *MoHTR2-*OX (Supplementary Tables 8 and 9). We found that 64.5% (240 genes) of the up-regulated genes in *MoHTR1*-OX were also up-regulated in *MoHTR2*-OX. Similarly, 36.6% (286 genes) of the down-regulated genes in *MoHTR1*-OX were also down-regulated in *MoHTR2*-OX (Supplementary Fig. 6). The percentages of EBE-containing DEGs in the promoter are 3.4% (40 genes out of 1,153 DEGs) in *MoHTR1*-OX and 12.3% (138 genes out of 1,115 DEGs) in *MoHTR2*-OX. Some of the not-affected genes contain EBE in the promoter, but the percentages are lower: 3.18% (1,383 genes) in *MoHTR1*-OX and 7.44% (3,237 genes) in *MoHTR2*-OX.

### Effect of MoHTR deletion on fungal development and virulence

**Fig. 5:**
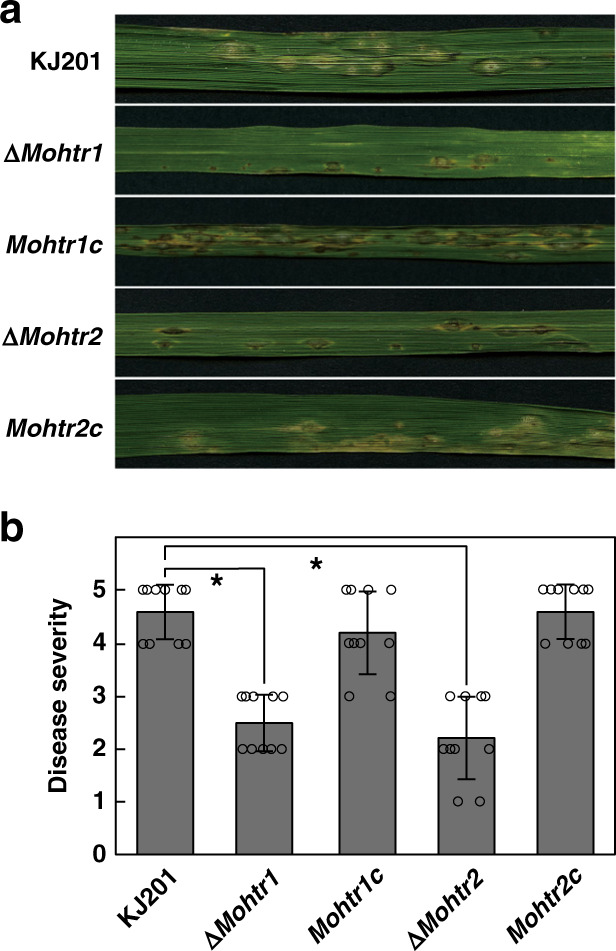
The requirement of *MoHTR1* and *MoHTR2* for full virulence. 4-week-old Nakdong rice seedlings were sprayed with conidial suspensions (5 × 10^4^ mL^−1^) of KJ201 (wild type), two *MoHTR* knockout mutants, and complemented mutant strains. **a** Representative infected leaves were collected at 7 dpi, and **b** quantified data are shown. *n* = 10 independently inoculated plants; mean ± SD; *P < 0.05, one-way ANOVA with Tukey’s HSD test. Three independent infection assays produced similar results. Detailed information about biological repeat experiments and statistical analysis underlying Fig. 5b are provided as a Source Data file.

We tested if MoHTR1 and MoHTR2 regulate fungal development by observing the vegetative growth, conidiation, conidial germination, and appressorium formation of Δ*Mohtr1* and Δ*Mohtr2*. We observed no significant differences between the mutants and KJ201 (Supplementary Table 10). However, the mutants exhibited significantly reduced virulence to rice. The average disease indices caused by Δ*Mohtr1* and Δ*Mohtr2* were 2.5 and 2.2, respectively, whereas that of KJ201 was 4.6 (Fig. 5). Rice sheaths were also inoculated for virulence assessment. The severity of invasion was quantified by rating the degree of infection progress (from 1 to 4) in over 100 infection sites (Supplementary Fig. 7). Invasive hyphae of KJ201 caused types 2–4 at 66.9% of the infection sites. However, Δ*Mohtr1* and Δ*Mohtr2* caused types 2–4 at 23.7% and 26.5% of the infection sites, respectively. Complemented strains of Δ*Mohtr1* and Δ*Mohtr2* displayed virulence level comparable to that of KJ201 (Supplementary Fig. 7). We also inoculated barley seedlings with these mutants to determine whether the function of these effectors is rice specific. Virulence of Δ*Mohtr1* and Δ*Mohtr2* was significantly reduced (Supplementary Fig. 8), indicating that their function is not rice specific.

### Expression of *MoHTR1* and *MoHTR2* in rice affects susceptibility to multiple pathogens

**Fig. 6:**
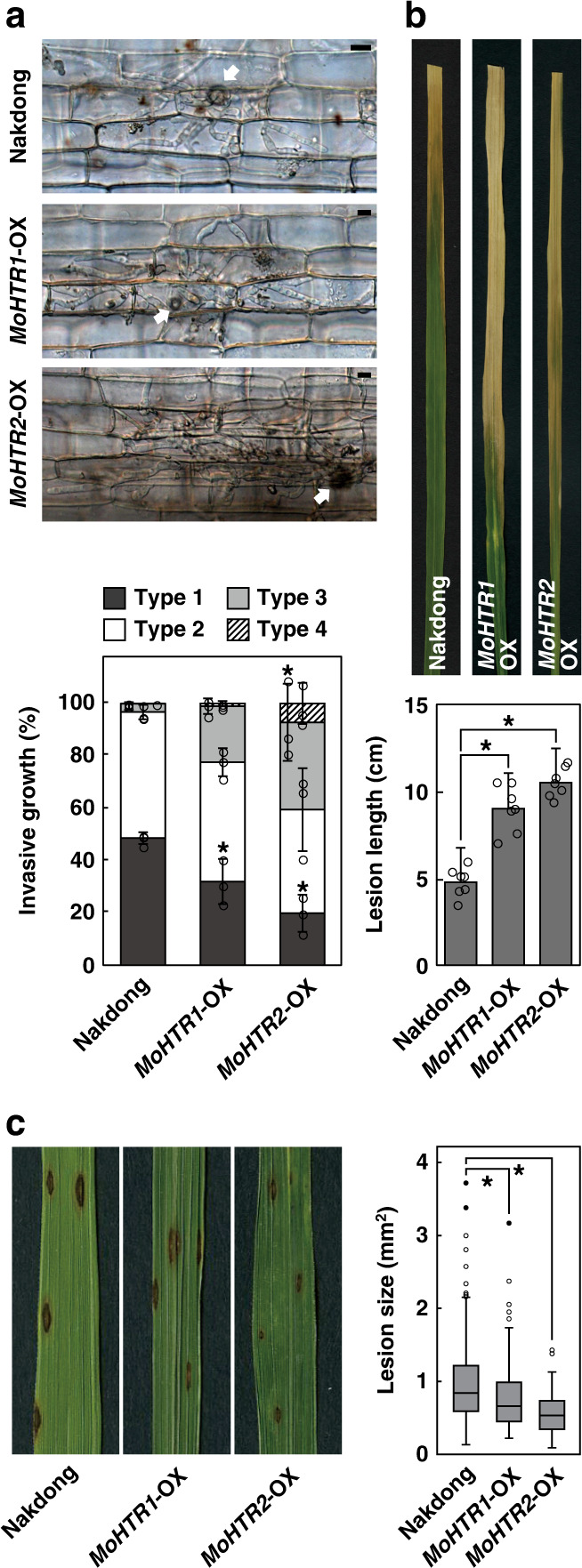
Expression of *MoHTR1* and *MoHTR2* in rice affects disease susceptibility to multiple pathogens. **a** Invasive growth of KJ201 in sheath cells of Nakdong, *MoHTR1*-OX, and *MoHTR2*-OX at 48 hpi was observed under light microscopy and rated (from type 1 to type 4 as described in the Methods). The arrow indicates the appressorium on initially penetrated cells. Each bar represents the proportion of individual infection types. *n* = 3 independent experiments; mean ± SD; *P < 0.05, one-way ANOVA (in randomized block design) with Tukey’s HSD test. Scale bars = 10 μm. **b** Representative disease symptoms caused by *X*. *oryzae* pv. *oryzae* (*Xoo*) at 7 dpi and lesion length. *n* = 7 independently inoculated plants; mean ± SD; *P < 0.05, one-way ANOVA with Tukey’s HSD test. **c** Representative disease symptoms caused by *C*. *miyabeanus* at 7 dpi and lesion size distribution. *n* = 349 for Nakdong, *n* = 143 for *MoHTR1*-OX, *n* = 95 for *MoHTR2*-OX; *P < 0.05, Kruskal-Wallis test with pairwise comparison. Boxes represent median, first, and third quartile. Whiskers show the highest and lowest values that are within 1.5 interquartile range. Three independent experiments produced similar results, and data from one experiment are presented. Detailed information about biological repeat experiments and statistical analysis are provided as a Source Data file.

We investigated whether *MoHTR1*-OX and *MoHTR2*-OX lines are more susceptible to *M*. *oryzae*. In sheaths of wild-type rice plants inoculated with KJ201, only 3.3% of the infection sites exhibited type 3 invasion at 48 hpi. However, in *MoHTR1*-OX and *MoHTR2*-OX, 22.4 and 35.2% of the infection sites, respectively, displayed type 3 or 4 at 48 hpi (Fig. 6a). We also determined whether the expression of *MoHTR1* or *MoHTR2* in rice affects susceptibility to other pathogens by infecting *MoHTR1*-OX and *MoHTR2*-OX lines with *Xanthomonas oryzae* pv. *oryzae* (*Xoo*), a hemibiotrophic bacterial pathogen that causes rice bacterial leaf blight, and *C*. *miyabeanus*, a necrotrophic fungal pathogen that causes rice brown spot. Both *MoHTR1*-OX and *MoHTR2*-OX infected with *Xoo* displayed more severe symptoms than wild-type plants (Fig. 6b). In contrast, necrotic lesions on both transgenic lines infected with *C*. *miyabeanus* were much smaller than those on wild type (Fig. 6c), suggesting that transcriptional reprogramming in rice by MoHTR1 and MoHTR2 alter rice resistance/susceptibility depending on the pathogen’s mode of infection.

## Discussion

Plant nuclei are the control hub for the immune system against diverse pathogens; signals generated upon the recognition of pathogens are conveyed to plant nuclei in which the signals are decoded into a cascade of molecular and cellular changes needed to execute appropriate defense responses^29^. Here, we show that *M. oryzae* delivers two nuclear effectors into rice nuclei to reprogram the expression of many immunity-associated genes. Besides enhancing susceptibility against *M. oryzae*, the reprogramming in gene expression by these effectors also significantly affected rice defense responses to other pathogens (Fig. 6), suggesting that the genes and processes modulated by the effectors are involved in defense against diverse pathogens. The discovery and functional validation of these novel nuclear effectors were accomplished via a series of experiments: (i) genome mining to identify potential nuclear effectors based on their sequence features associated with protein secretion and DNA binding; (ii) RNA-seq and protein structural modeling analyses to evaluate if their expression is induced during infection and characterize the structure of their DNA-binding domain, respectively; (iii) live-cell imaging of fluorescently-tagged MoHTRs to demonstrate their secretion and translocation into rice nuclei during infection; (iv) protein binding microarray and yeast one-hybrid assays to identify the effector binding element for each MoHTR; (v) both transient and stable expressions of each MoHTR in rice to demonstrate their regulation of the transcription of immunity-associated genes; and (vi) evaluation of how transgenic rice lines expressing each MoHTR respond to bacterial and fungal pathogens that employ different modes of infection.

Plant pathogens deliver cytoplasmic effectors into host cells via several specialized systems. Bacterial pathogens directly inject their effectors using the type III secretion system^30^, and oomycete pathogens deliver effectors by a system that involves host targeting signals^31^. Fungal pathogens have been shown to secrete cytoplasmic effectors via multivesicular bodies or focal membrane-rich structure^32,33^. Cytoplasmic effectors of *M. oryzae* are secreted to the BIC via an unconventional secretory pathway, involving exocyst and SNARE proteins, and translocated into the host cytoplasm^34^. Live-cell imaging of fluorescently-tagged MoHTR1 and MoHTR2 clearly showed that both effectors were secreted through BIC to enter rice cells and subsequently migrated into rice nuclei (Figs. 1 and 2). These effectors contain predicted NLSs that are distinct from those present in known nuclear effectors. While nuclear localization of TAL and SP7 effectors was observed in multiple organisms^35,36^, MoHTR1-Δsp:RFP and MoHTR2-Δsp:RFP moved to rice nuclei but not *M. oryzae* nuclei (Supplementary Fig. 3), suggesting that MoHTR1 and MoHTR2 may require host factors for moving into the nucleus. However, the mechanism underpinning their nuclear localization still remains to be characterized. Similar to an earlier report showing the movement of PWL2:tdTomato, a fluorescent protein-tagged *M*. *oryzae* cytoplasmic effector, through plasmodesmata (PD)^11^, MoHTRs also appear to translocate into the nuclei of neighboring cells, presumably via PD, before the hyphal invasion.

We identified EBEs for MoHTR1 (CAATCTTC) and MoHTR2 (CCACCTCC) in the rice genome (Fig. 3). Each nucleotide substitution in EBE significantly reduced DNA binding affinities, consistent with their sequence-specific binding. Although we focused on characterizing two EBEs, they are unlikely to be the only target sequences for MoHTRs. Other potential EBE candidates still exhibited strong binding affinities (Supplementary Table 2). Since it is not yet clear if the binding affinity correlates with the degree of suppression in gene expression, the possibility that these effectors also target those genes with other EBEs warrants further studies. Our approach to identifying rice genes potentially targeted by these effectors was not only based on the presence of EBE in the promoter region but also differential expression during *M*. *oryzae* infection. Using these criteria, we could efficiently identify candidate target genes associated with rice immunity, including previously reported defense/resistance-related genes^26–28^.

Transcripts of the genes targeted by MoHTR1 and MoHTR2 increased in rice plants infected by Δ*Mohtr1* and Δ*Mohtr2*, while their expression was reduced in *MoHTR*-OX lines (Fig. 4). These results support the hypothesis that MoHTR1 and MoHTR2 function as repressors of target gene expression. A transactivation assay in rice protoplasts suggests that MoHTR1 and MoHTR2 do not have an intrinsic repressor activity (Supplementary Fig. 4). One possibility is that these effectors may suppress the expression of their targets by interfering with the binding of rice transcription activators through competition for the same binding sites. It is also possible that they may interact with transcription repressor(s) to exert their effect. In addition to directly repressing specific target genes, MoHTR1 and MoHTR2 also appear to modulate the expression of many other immunity-associated genes, including hormone signaling and basal defense genes, indirectly (Supplementary Fig. 5). These data, together with RNA-seq analysis of *MoHTR*-OX lines, strongly suggest that MoHTR-mediated transcriptional reprogramming likely causes broad changes in rice immunity. Although whether MoHTR1 and MoHTR2 affect immunity-related gene expression in barley remains unknown, the reduced virulence of Δ*Mohtr1* and Δ*Mohtr2* in barley (Supplementary Fig. 8) suggests that they also reprogram such genes in barley. A comparative gene expression analysis between rice and barley after infecting with Δ*Mohtr1* and Δ*Mohtr2* likely reveals similarities and differences in how these cereals defend against *M. oryzae*.

Consistent with the genome-wide transcriptional reprogramming by MoHTR1 and MoHTR2, the resulting effect on rice immunity went beyond defense against *M. oryzae*. Transgenic expression of *MoHTR1* and *MoHTR2* in rice increased susceptibility to *M*. *oryzae* and *Xoo*, hemibiotrophic pathogens, but enhanced resistance to a necrotrophic pathogen, *C*. *miyabeanus* (Fig. 6). Further studies are needed to understand how the overexpression of these two nuclear effectors with disparate binding sites similarly affected rice defense against three different pathogens. However, we can speculate a likely mechanism. Plants rely on common defense machineries against different pathogens rather than employing pathogen-specific machineries because evolving defense systems customized for individual pathogens is not possible. This is why although different pathogens have evolved a wide variety of strategies and effectors to dismantle or suppress host defense, they often target similar genes and processes. Both MoHTR1 and MoHTR2 affected the expression of several immunity-associated genes such as some PR genes and phytohormone signaling genes (Supplementary Fig. 5). In addition, 64.5% (240 genes) of the up-regulated genes in *MoHTR1*-OX rice were also up-regulated in *MoHTR2*-OX. Similarly, 36.6% (286 genes) of the down-regulated genes in *MoHTR1*-OX rice were also down-regulated in *MoHTR2*-OX rice (Supplementary Fig. 6). Similar rice gene expression patterns resulting from overexpressing *MoHTR1* and *MoHTR2* likely underpin similar effects on resistance/susceptibility to multiple pathogens.

The ambivalent immunity to multiple pathogens caused by MoHTRs highlighted a critical consideration in developing disease management strategies through targeted gene manipulations. Considering the likelihood that multiple pathogens simultaneously attack rice in the field, the overall plant health is likely to be determined by which combinations of pathogens invade rice. Genetic manipulations of resistance/susceptibility genes to increase resistance against one pathogen may inadvertently create a double-edged sword to plant health by creating unintended susceptibility to other pathogens. This ambivalent immunity and underlying mechanism should be investigated to understand complex plant–pathogen interactions in nature and apply this understanding to develop durable resistance against multiple pathogens.

Our work demonstrates that *M. oryzae* employs nuclear effectors to reprogram the transcription of immunity-associated genes in rice and that some of them likely function in other plants. Characterization of how these fungal nuclear effectors modulate host immunity will help answer multiple questions crucial for understanding the mechanism of plant–pathogen interactions, such as how fungi evade host defense, which plant genes are needed for defense against specific pathogens, and how such plant genes work together or antagonize in responding to diverse pathogens. This understanding should help guide the development of effective strategies for managing plant diseases of economic or environmental significance.

## Methods

### Genome mining to identify nuclear effector candidates and identification of their target genes in rice

Nuclear effector candidates were mined from the genome of *M. oryzae* strain 70–15^37^ archived in Comparative Fungal Genomics Platform 2.0 (CFGP2.0; http://cfgp.snu.ac.kr)^18^. Secreted proteins and transcription factors of *M*. *oryzae* were curated in Fungal Secretome Database (FSD; http://fsd.snu.ac.kr)^12^ and Fungal Transcription Factor Database (FTFD; http://ftfd.snu.ac.kr)^13^, respectively. BLASTMatrix, a tool in CFGP2.0^18^, was used to search the homologs of MoHTR effectors in the genomes of diverse species and different *M*. *oryzae* isolates. We used WoLF-PSORT^14^, NLStradamus^15^, and cNLS mapper^16^, to check the presence of potential nuclear localization signal(s) in MoHTR effectors.

We collected 1-kb upstream sequences from the coding region of all rice genes from the Rice Annotation Project Database (RAP-DB)^38^ and checked the presence of each EBE. Differentially expressed genes (DEGs) at 36 hpi (biotrophic stage) were identified using transcriptome data generated from infected rice sheaths^17^. Gene ontology (GO) enrichment analysis was performed using RiceNetDB^25^. Information related to previously characterized genes was collected from the funRiceGenes Database^39^.

### Assays for the subcellular location of MoHTR proteins

A fragment containing the 1-kb upstream and coding regions of each MoHTR gene was inserted to pFPL-Rh vector^40^ for MoHTR:mRFP expression. Plasmids containing PWL2pro:PWL2:eGFP:NLS and BAS4pro:BAS4sp:eGFP^11^ were used to label plant nuclei and EIHM compartment, respectively. After inoculating rice sheaths with fungal transformants, the location of each protein was microscopically imaged. pFPL-Rh vectors that express each MoHTR-Δsp:mRFP under control of the *Fusarium verticillioides* EF1α promoter were used to investigate the localization of individual MoHTRs in fungal cells. Fungal transformants expressing these recombinant proteins were generated via PEG-mediated transformation. Their expression was confirmed by microscopically observing the fluorescent signal in the conidia of individual transformants. *MoHTR1*-Δsp and *MoHTR2*-Δsp were cloned into pENTR^TM^/D-TOPO (Invitrogen) to determine their localization in the rice cytoplasm. After confirming the presence of each insert via sequencing, these constructs were moved to the destination vector p7FGW2 for C-terminal GFP fusion^41^ using the Gateway LR clonase (Invitrogen). The primers used for fungal transformation are listed in Supplementary Table 11. The resulting constructs were introduced into rice mesophyll protoplasts using PEG-calcium mediated transformation method^42^. OsABF1:mRFP was used to label plant nuclei^23^.

Confocal laser scanning microscope LSM710 (Carl Zeiss) with C-Apochromat 40×/1.20 W Korr M27 water immersion objective was used for imaging. The excitation/emission wavelengths for eGFP and mRFP were 488/492–562 nm and 543/590–725 nm respectively, and the pinhole setting for emission fluorescence was 2 airy units. The Axio Imager A1 microscope (Carl Zeiss) was used to acquire epifluorescence and differential interference contrast (DIC) images. AxioCam HRc camera and Axiovision version 4.8 were used for image acquisition. The light source was HBO 100, a 100-W high-pressure mercury plasma arc-discharge lamp.

### Protein binding microarray to identify MoHTR-binding DNA sequences

The DNA microarray used contains 232,145 probes that consist of quadruple 9-mer oligonucleotides and cover all possible 9-mer sequences (Agilent Technology). Tandemly repeated 9-mers were followed by 5′-CGGAGTCACCTAGTGCAG-3′ and a 5-nucleotide thymidine linker to the chip^24^. Each DsRed:6× His-linked MoHTR-Δsp was expressed using pET-32a vector (Novagen) in *Escherichia coli* strain BL21-CodonPlus (Stratagene). Recombinant proteins were purified using TALON metal affinity resins (Clontech). Protein binding to the microarray chip was performed in the reaction solution [200 nM protein in PBS, 2% BSA, 50 ng/μl salmon-testes DNA (Sigma) and 50 μM zinc acetate]^24^. The fluorescent signal resulting from MoHTR-Δsp-DNA probe interaction was detected using Genepix4000B microarray scanner (Molecular Devices). Multiple orders of k-mer (k ≤ 9) repeatedly appear in the DNA microarray chip because each 9-mer sequence is quadrupled. There are 32,768 combinations of 8-mer nucleotides that repeatedly appear in 36 different probes. We compared the median intensity of probes containing a particular 8-mer to identify the strongest effector binding element for each effector.

### Validation of MoHTR-DNA interaction in vivo

For yeast one-hybrid, the EBE-centered 38 bp promoter fragment from each rice gene was inserted into the minimal promoter of yeast cytochrome C1 (CYC1) gene in pLacZi (Clontech). The fragments used correspond to the −337 to −299 region from the start codon of *OsMYB4* and the −766 to −728 region of *OsWRKY45*. Because there are two binding sites for *OsHPL2*: −875 to −837 and −745 to −707 from the start codon, both sequences were used for analysis. The β-galactosidase reporter was placed under each modified *CYC1* promoter containing a MoHTR-target site. Each reporter vector was introduced to yeast strain YM4271 via LiAc-mediated transformation, and transformants were selected on SD-ura medium. A transformant without reporter auto-activity was used as a control strain. Each *MoHTR*-Δsp was inserted into pDEST22 (Invitrogen) to fuse it with the sequence encoding the GAL4 activation domain. The recombinant vectors were introduced to the control strains. Transformants were selected on SD-ura-trp medium and transferred to YPD agar supplemented with 0.004% X-gal (W/V). Blue staining resulting from DNA–protein interaction was checked after 5 days of incubation.

For an in planta interaction assay, reporter vectors containing the luciferase (LUC) gene under the *OsMYB4* and *OsWRKY45* promoters were generated. We constructed pADT (for Activation Domain Tag) by inserting a quadrupled activation domain (AD) of the *Xoo* TAL effector AvrXa10 at the downstream of the gateway cassette in pH2GW7^41^. Each *MoHTR*-Δsp was integrated in pADT using LR clonase (Invitrogen). The CaMV 35S promoter was used to express each *MoHTR*-Δsp linked to AD. The control vector that contains the beta-glucuronidase (GUS) gene under the maize ubiquitin promoter was used for internal control (Fig. 3d). The effector–DNA interaction was quantified by measuring luminescence after co-transfecting the effector, reporter, and internal control vectors into rice protoplasts. Normalized luminescence (luminescence/GUS activity) resulting from effector expression was compared with that of the empty vector control.

### Transcriptional activity assay in rice protoplasts

To evaluate the intrinsic transcriptional activity of each MoHTR, a *LUC* reporter was expressed under the control of the basal expression promoter containing 5× GAL4 upstream activation sequence (UAS) and minimal 35S promoter (35S_mini_)^43^. The effector vector was designed to produce MoHTR-Δsp fused with GAL4 DNA-binding domain (GBD) for interaction with the 5× UAS (Supplementary Fig. 4). To evaluate the transcriptional activity of each MoHTR at the target gene promoters, a *LUC* reporter was expressed under the control of each target gene promoter. The effector vector was designed to produce MoHTR-Δsp using the CaMV 35S promoter and the TMV translation enhancer (Ω) sequence^44^ (Fig. 4a). The control vector that contains the GUS gene under the maize ubiquitin promoter was used for internal control.

The reporter, effector, and control vectors were introduced into rice protoplasts isolated from 2-week-old etiolated rice seedlings using a published protocol^42^ with slight modification. Transfected protoplasts were incubated for 4 hours in W5 solution (153 mM NaCl, 125 mM CaCl_2_, 5 mM KCl, 2 mM MES), and their lysate was harvested using lysis buffer [25 mM Tris phosphate (pH 7.8), 2 mM diaminocyclohexane tetraacetic acid, 10% glycerol, 1% Triton X-100, 2 mM dithiothreitol]. A LUC reaction with the lysate was performed using the Luciferase Assay System from Promega. GUS assay using the cell lysate of transfected protoplasts was conducted in the reaction solution [10 mM Tris-Hcl (pH 8.0), 2 mM MgCl_2_, 1 mM 4-Methylumbelliferyl-ß-D-glucuronide]^45^. The signal was quantified by the VICTOR3 multilabel plate reader (PerkinElmer). Normalized LUC activity was calculated by dividing the luminescence intensity by the GUS activity. The relative transcriptional activity of MoHTR was measured by comparing it with normalized LUC activity of the control reaction.

### Genetic manipulation of *M*. *oryzae*

*M*. *oryzae* field isolate KJ201 was obtained from the Center for Fungal Genetic Resources at Seoul National University. Four-day-old mycelia in complete medium broth were collected and treated with 3.3% lysing enzyme in 20% sucrose solution (w/v). After suspending generated protoplasts in STC [20% sucrose (w/v), 50 mM Tris-HCl, 50 mM CaCl_2_], they were used for PEG-mediated transformation. Knockout mutants were produced by replacing each target gene with a hygromycin phosphotransferase (HPH) cassette amplified from pBCATPH^46^. Double-joint PCR was used to attach the 5′ and 3′ flanking regions of each target gene to the HPH cassette. Fungal transformants were selected using 200 ppm hygromycin B. Gene deletion was confirmed by priority-based direct PCR^47^ and Southern analysis. Each gene with its own promoter was reintroduced to the corresponding knockout mutant using PEG-mediated transformation for complementation. A geneticin resistance gene cassette used for complementation was amplified from pII99^48^. The complemented strains were selected on TB3 agar medium containing 800 ppm geneticin. The primers used for fungal gene manipulation are listed in Supplementary Table 11.

### Production of transgenic rice plants expressing each MoHTR gene without the signal peptide

*MoHTR1*-Δsp and *MoHTR2*-Δsp were amplified by PCR using the primers listed in Supplementary Table 11 and then integrated into the binary vector pH2GW7^41^ using the Gateway LR clonase (Invitrogen). The resulting constructs were introduced into the rice cultivar Nakdong via *Agrobacterium tumefaciens*-mediated transformation. LBA4404 harboring each construct was cultured in AB medium (K_2_HPO_4_ 3 g L^−1^, NaH_2_PO_4_ 1 g L^−1^, NH_4_Cl 1 g L^−1^, MgSO_4_ 0.3 g L^−1^, KCl 0.15 g L^−1^, CaCl_2_ 7.5 mg L^−1^, FeSO_4_ 2.5 mg L^−1^) supplemented with 10 mg L^−1^ streptomycin and 50 mg L^−1^ hygromycin B for 3 days at 28 °C. Transgenic calli were selected on a medium containing 50 mg L^−1^ hygromycin B and 250 mg L^−1^ cefotaxime. Rice plants were grown in a greenhouse under the 14/10-h light/dark period, 24–28 °C, and 70–80% humidity.

### Gene expression analysis via quantitative RT-PCR and RNA-Seq

Total rice RNA was isolated using the Easy-spin total RNA extraction kit (iNtRON Biotechnology) according to the manufacturer’s instructions. First-strand cDNAs were synthesized using 2 μg of total RNA and the ImProm-II Reverse Transcription System (Promega) with oligo (dT) primers. qRT-PCR reactions were performed using a Rotor-Gene Q real-time PCR cycler (Qiagen) and the gene-specific primers listed in Supplementary Table 11. Each PCR tube contained 5 μl of 2× Rotor-Gene SYBR Green PCR master mix (Qiagen), 25 ng of cDNA, and 15 pmol of each primer. The thermal cycling conditions were 10 min at 94 °C followed by 40 cycles of 15 s at 94 °C and 1 min at 60 °C.

The quality of extracted RNA samples was assessed using Bioanalyzer 2100 (Agilent). RNA-Seq libraries were prepared using the TruSeq RNA Library Prep Kit (Illumina) and sequenced using HiSeq2500 (Illumina). Raw sequence reads were trimmed to remove adaptor sequences, and those with a quality lower than 20 were removed using the NGS QC Toolkit v2.3.3^49^. All reads were assembled and mapped via the use of HISAT2 v2.1.0^50^ and StringTie v1.3.5^51^. DEGs in the *MoHTR*-OX lines were identified as the genes whose transcript abundance was ≥ 2-fold higher or ≤ 0.5-fold lower than their transcripts in Nakdong rice.

### Growth and developmental characteristics of *MoHTR* mutants

Vegetative growth was checked by recording the diameter of each culture on complete agar medium (CM; 0.2% peptone, 1% glucose, 1% casamino acid, 0.1% yeast extract, 0.15% KH_2_PO_4_, 0.05% KCl, 0.6% NaNO_3_, 0.05% MgSO_4_, 0.1% trace element, 0.1% vitamin supplement, and 1.5% agar, pH 6.5) and minimal agar medium (MM; 0.15% KH_2_PO_4_, 0.05% KCl, 0.6% NaNO_3_, 1% glucose, 0.05% MgSO_4_, 0.1% trace element, 0.1% vitamin supplement, and 1.5% agar, pH 6.5). Conidiation was estimated by counting the number of conidia produced by 6-day-old cultures on V8 agar in 6-well culture plate (SPL). All cultures were grown at 25 °C under constant light. Conidial germination and appressoria formation were quantified at 12 hours after spotting a conidial suspension on hydrophobic coverslip.

### Infection assays for fungal and bacterial pathogens

The rice cultivar Nakdong was grown in a growth chamber at 28 °C and 80% humidity under a 16 h-light/8 h-dark photoperiod. After spray-inoculating 4-week-old plants with an *M*. *oryzae* conidial suspension (5 × 10^4^ conidia/mL in 250 ppm Tween20), the inoculated rice plants were incubated for 7 days. A numerical scoring system was employed to quantify disease: type 0 (no visible infection) to type 5 (large eyespot lesions, approximately 5 mm in length)^52^.

Six-week-old rice leaf sheaths were inoculated with an *M*. *oryzae* conidia suspension (2 × 10^4^ conidia/mL in sterile water). Inner epidermal layers of infected sheaths were excised and observed using a microscope. The disease severity was classified into four types based on the level of invasive fungal growth. Type 1 indicates that invasive hyphae remain in initially penetrated rice cells. Type 2 is defined as those that show the movement of invasive hyphae in initially infected cells to adjacent cells. In types 3 and 4, invasive hyphae move further to the surrounding layers of cells from the previous type (Supplementary Fig. 7).

Barley seedlings were grown in a growth chamber at 28 °C and 80% humidity under a 16 h-light/8 h-dark photoperiod. *M*. *oryzae* conidial suspension (5 × 10^4^ conidia/ml in 250 ppm Tween20) was sprayed to 10-day-old seedlings. The resulting lesions were observed and quantified after 5 days.

Five-week-old rice leaves were infected with *Xoo* K2, a strain compatible with cultivar Nakdong. The suspension of *Xoo* cells used for infection was prepared from colonies grown on nutrient agar at 28 °C for three days. Rice leaves were inoculated with a bacterial suspension adjusted to OD_600_ 0.7 using the scissor clip method^53^. Lesion length was measured at 7 dpi.

Conidia of *C*. *miyabeanus* strain Cm36 were collected from 10-day-old culture on PDA. A conidial suspension (1 × 10^3^ conidia/ml) was prepared using 250 ppm Tween20. Spray inoculated rice plants were incubated at 30 °C and 85% humidity. Infected leaves were collected after 5 days. The lesion size was quantified using ImageJ^54^.

### Reporting summary

Further information on research design is available in the Nature Research Reporting Summary linked to this article.

## Supplementary information

Supplementary Information

Peer Review File

Reporting Summary

## Data Availability

Data supporting the findings of this work are available within the paper and its Supplementary Information files. A reporting summary for this Article is available as a Supplementary Information file. The datasets and plant materials generated and analyzed during the current study are available from the corresponding author upon request. RNA-seq data has been deposited into the NCBI Sequence Read Archive under accession number PRJNA668035. Source data are provided with this paper.

## Source data

Source Data
