# Supplementary material for: Two nuclear effectors of the rice blast fungus modulate host immunity via transcriptional reprogramming
Source: Nat Commun. 2020 Nov 17;11:5845. doi: 10.1038/s41467-020-19624-w (PMC7672089; doi:10.1038/s41467-020-19624-w)
Supplement: Supplementary file 1 — Supplementary Information [file 41467_2020_19624_MOESM1_ESM.pdf]

Two nuclear effectors of the rice blast fungus modulate host immunity *via* transcriptional reprogramming

Kim *et al.*

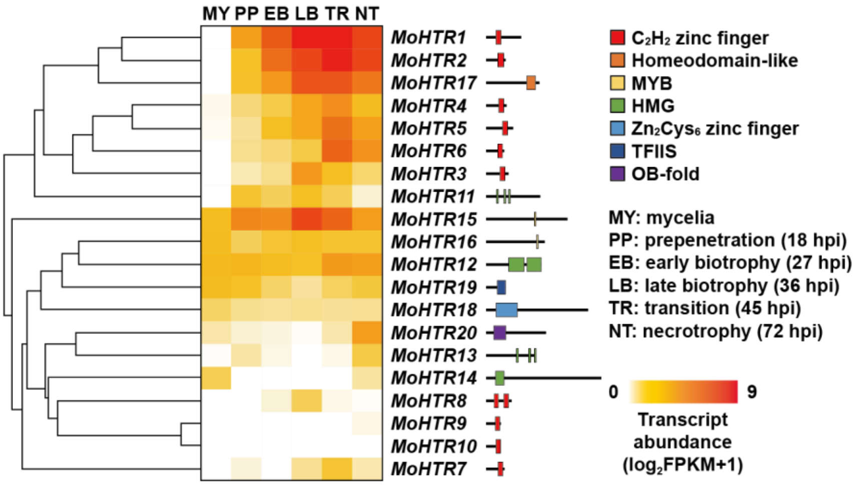

Supplementary Figure 1. Transcription profiling of 20 *MoHTR* genes under multiple conditions and the domain structure of their products.

The abundance of *MoHTR* transcripts during multiple stages of infection, including the prepenetration (PP), early biotrophy (EB), late biotrophy (LB), transition (TR), necrotrophy (NT), and mycelial (MY) stages, was calculated as $\log_2$(FPKM+1) from RNA-Seq data[17]. Their expression patterns were analyzed *via* hierarchical clustering of the data using the Euclidean distance matrix. The heat map shows transcript abundance. Different types of DNA binding domains were noted using different colors.

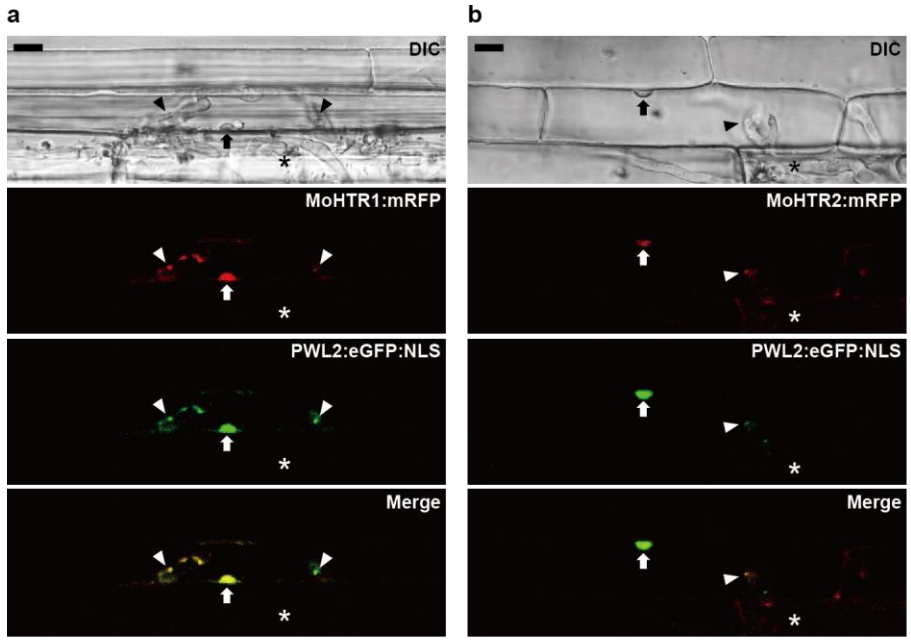

Supplementary Figure 2. Localization of MoHTR1 and MoHTR2 in rice nuclei during infection.
DIC and confocal images of rice sheaths infected by *M. oryzae*. Invasive hyphae (arrowheads) moved to adjacent cells from the initially infected cell (asterisks) at 40 hpi. (a) MoHTR1:mRFP and (b) MoHTR2:mRFP co-localized with PWL2:eGFP:NLS, a marker used to label rice nuclei (arrows). Scale bar = 10 μm. Representative micrographs are shown from three independently infected rice sheaths and additional representative data are provided as a Source Data file.

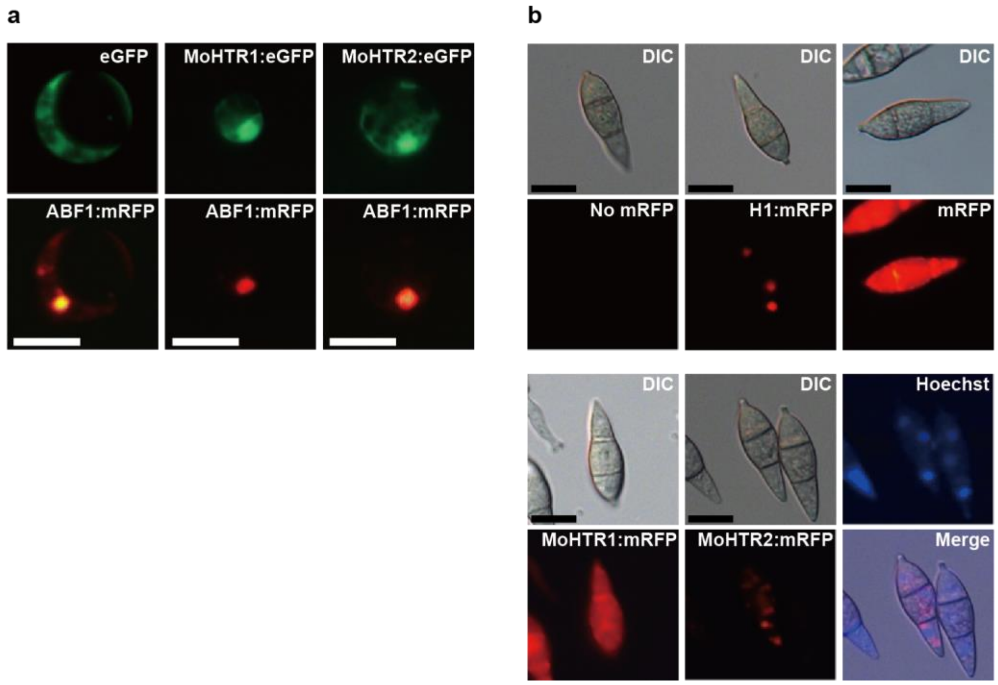

Supplementary Figure 3. Subcellular locations of MoHTR1-Δsp and MoHTR2-Δsp in rice and *M. oryzae*.
(a) Cellular location of MoHTR1-Δsp:eGFP and MoHTR2-Δsp:eGFP in rice protoplasts (top panel). MoHTR-Δsp:eGFP was expressed using the CaMV 35S promoter. OsABF1:mRFP was used to label rice nuclei (bottom panel). Scale bar = 10 μm. (b) Location of MoHTR1-Δsp:mRFP and MoHTR2-Δsp:mRFP in *M. oryzae* conidia. MoHTR-Δsp:mRFP was expressed using the EF1α promoter. H1:mRFP and Hoechst staining mark fungal nuclei. Scale bar = 10 μm. Representative micrographs are shown from three independent experiments and additional representative data are provided as a Source Data file.

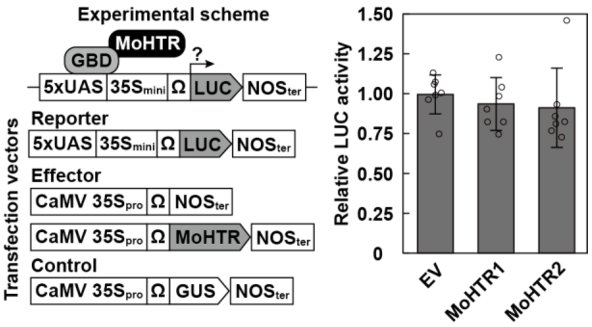

Supplementary Figure 4. Intrinsic transcriptional activity of MoHTR1 and MoHTR2. The basal-level expression of LUC was regulated by the minimal CaMV 35S promoter ($35S_{mini}$) and translation enhance sequence (Ω). Tandem repeats of the GAL4 upstream activation sequence (5×UAS) were placed in front of the $35S_{mini}$ to recruit proteins containing the GAL4 DNA-binding domain (GBD). Relative LUC activities in rice protoplasts transfected with a *MoHTR1*-Δsp or *MoHTR2*-Δsp expressing vector was compared with those transfected with an empty vector. Bars are mean ± SD (n=7 independently transfected protoplasts). Experiments with three biological replicates resulted in similar results. Detailed information about biological replicates and statistical analysis are provided as a Source Data file.

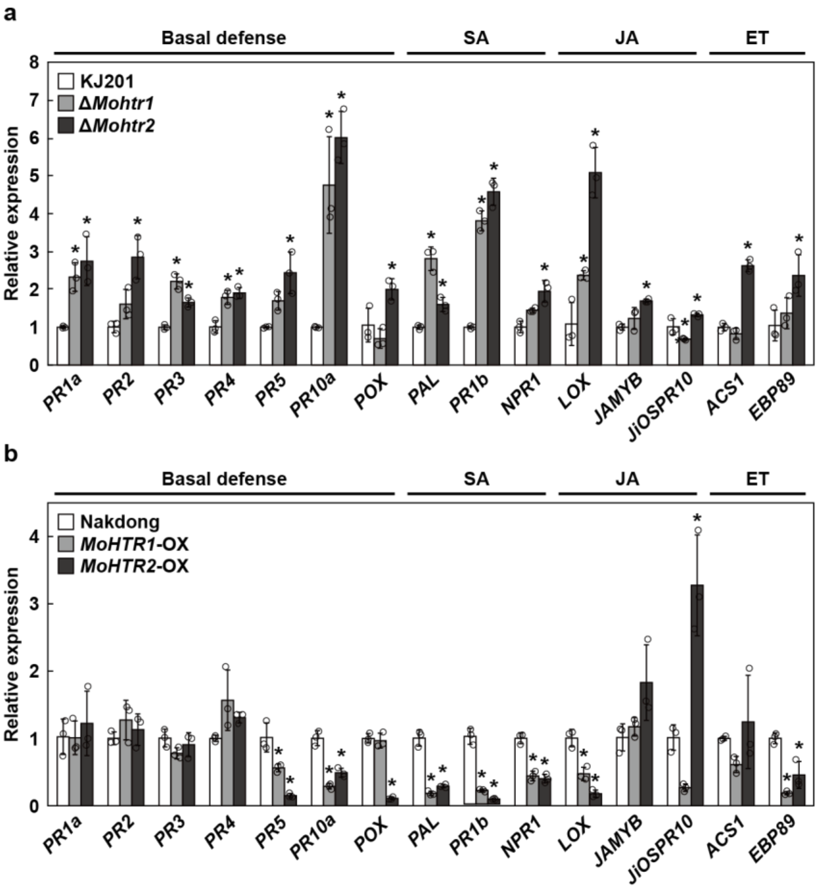

Supplementary Figure 5. Rice immunity-associated genes affected by *MoHTR1* and *MoHTR2.*

The abundance of transcripts from rice immunity-associated genes was quantified using qRT- PCR. (a) Expression levels of rice basal defense and hormone signaling-related genes in leaves of cultivar Nakdong infected by Δ*Mohtr1* and Δ*Mohtr2* were compared with those in Nakdong infected with KJ201. (b) Expression levels of the same rice genes in two *MoHTR*-OX lines were compared with those in Nakdong. Mean ± SD, three independent experiments; *$P < 0.05$, one-way ANOVA with Tukey's HSD test. Detailed information about biological replications and statistical analysis are provided as a Source Data file.

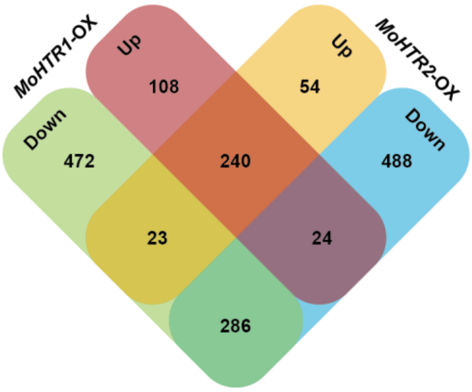

Supplementary Figure 6. Comparison of DEGs in *MoHTR1*-OX and *MoHTR2*-OX.
The Venn diagram shows the numbers of genes that are up-regulated ($\log_2(FPKM_{MoHTR\text{-}OX}+1)/(FPKM_{control}+1) > 1$) and down-regulated ($\log_2(FPKM_{MoHTR\text{-}OX}+1)/(FPKM_{control}+1) < -1$) in *MoHTR1*-OX and *MoHTR2*-OX lines.

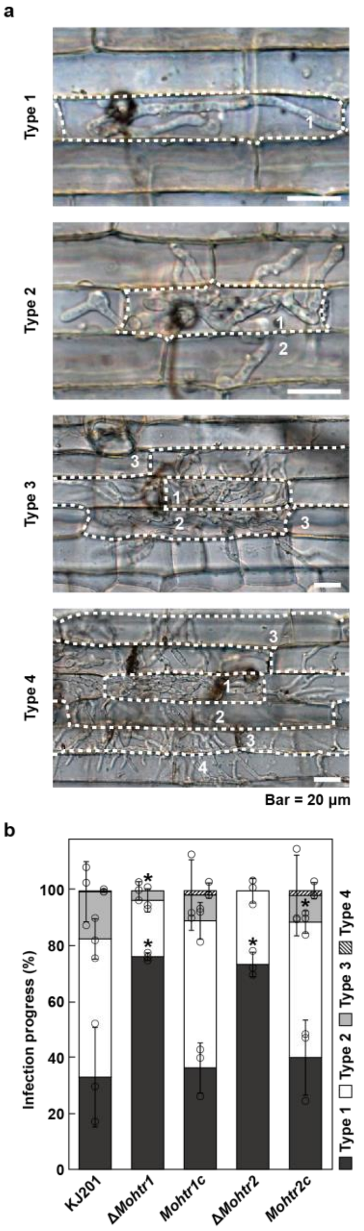

Supplementary Figure 7. The crucial role of *MoHTR1* and *MoHTR2* during the early stage of infection.
(a) Representative images for infection types 1-4. The severity of invasive growth is rated from type 1 to type 4. Numbers denoted the position of cells surrounding the initially infected cell (designated as 1). Type 1 indicates single-cell invasion. Invasive hyphae in the initially infected cell migrate to immediately adjacent cells (designated as 2) in type 2. In types 3 and 4, fungal hyphae migrate further to the cells at 3 and 4 positions, respectively. (b) Compatible rice leaf sheaths were infected with KJ201, Δ*Mohtr1*, Δ*Mohtr2*, and the complemented mutant strains. After 48 hpi, appressorial penetration sites (over 100 sites) were rated as type 1 to 4 according to the degree of infection progress. Each bar represents the proportion of individual infection types. $n = 3$ independent experiments; mean ± SD; $*P < 0.05$, one-way ANOVA (in randomized block design) with Tukey's HSD test. Detailed information about biological repeat experiments and statistical analysis of Supplementary Figure 7b are provided as a Source Data file.

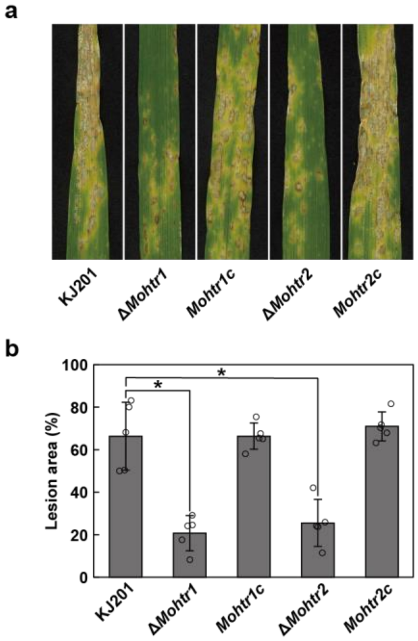

Supplementary Figure 8. Role of *MoHTR1* and *MoHTR2* on barley infection.
10-day-old barley seedlings were sprayed with conidial suspensions (5×10^4 mL^{-1}) of KJ201, two *MoHTR* knockout mutants, and complemented mutant strains. (a) Representative infected leaves were collected at 5 dpi and (b) quantified data are shown. $n = 5$ independently inoculated plants; mean ± SD; $^*P < 0.01$, one-way ANOVA with Tukey's HSD test. The experiments were performed three times. Detailed information on biological repeat experiments and statistical analysis of Supplementary Figure 8b are provided as a Source Data file.

Supplementary Table 1. Host transcription reprogramming effector candidates.

Locus number	Gene name	DNA binding domain[a]	Secretion signal[b]		NLS[c]	Protein size
			Cleavage site	Probability		
MGG_10276T0	*MoHTR1*	C_2H_2 zinc finger	19~20	0.998	P	198
MGG_10280T0	*MoHTR2*	C_2H_2 zinc finger	18~19	0.999	N	110
MGG_04580T0	*MoHTR3*	C_2H_2 zinc finger	19~20	0.999	-	126
MGG_05518T0	*MoHTR4*	C_2H_2 zinc finger	24~25	0.896	C	116
MGG_00916T0	*MoHTR5*	C_2H_2 zinc finger	19~20	0.999	N, P	154
MGG_04546T0	*MoHTR6*	C_2H_2 zinc finger	19~20	1	P	103
MGG_07699T0	*MoHTR7*	C_2H_2 zinc finger	19~20	0.999	P	105
MGG_15926T0	*MoHTR8*	C_2H_2 zinc finger	19~20	0.998	-	143
MGG_10556T0	*MoHTR9*	C_2H_2 zinc finger	19~20	0.996	P	83
MGG_17301T0	*MoHTR10*	C_2H_2 zinc finger	19~20	0.998	P	82
MGG_14836T0	*MoHTR11*	High mobility group	19~20	0.998	N	304
MGG_04374T0	*MoHTR12*	High mobility group	18~19	0.739	N	309
MGG_13742T0	*MoHTR13*	High mobility group	20~21	0.998	N, C, P	281
MGG_17098T0	*MoHTR14*	High mobility group	19~20	0.89	N, P	644
MGG_10494T0	*MoHTR15*	Myb	15~16	0.99	P	456
MGG_06434T0	*MoHTR16*	Myb	32~33	0.968	C, P	329
MGG_09628T0	*MoHTR17*	Homeodomain-like	22~23	0.973	P	299
MGG_12097T0	*MoHTR18*	Zn_2Cys_6	25~26	0.649	-	572
MGG_03072T0	*MoHTR19*	Transcription factor TFIIS	16~17	0.571	-	110
MGG_13241T0	*MoHTR20*	Nucleic acid-binding, OB-fold	18~19	0.997	P	336

[a]Domain prediction results were retrieved from FTFD[13].

[b]Their secretion signals were retrieved from CFGP2.0[18].

[c]WoLP-PSORT[14] (P), NLStradamus[15] (N), and cNLS mapper[16] (C) were used to predict NLS.

Supplementary Table 2. Effector binding element candidates of MoHTR1 and MoHTR2.

EBE candidates	Median signal intensity
**MoHTR1**	
CAATCTTC	27907.5
CTCATACC	25628
CTCAACTC	24737.5
TCTCAATC	24106.5
CCACCTCC	23987.5
TCAATCTC	23949
CCTCCACC	23944.5
TCCAATCC	22897
CTAACTCC	22505.5
AACTCCTC	22094
CTCTAATC	21793.5
CCTCCTCC	21694
AACTCTCC	21630
CTTCAATC	21084
CCAACTCC	20796
CAATCTCC	20785
CAATCCTC	20117
CCTCAATC	19738
CATACTCC	19143
CTCCAACC	14822
CCATACAC	14056.5
CCGCCACC	13363.5
**MoHTR2**	
CCACCTCC	11796
CCTCCACC	11567.5
CCTCCTCC	10775.5
CCTCCGCC	9496.5
CCGCCTCC	8800
CATACCTC	8485.5

Supplementary Table 3. Candidate target genes for MoHTR1.

Locus number	Annotation	Gene name	Function	Expression*
Os01g0615100	Similar to substilin /chymotrypsin-like inhibitor.	*OCPI1*	Grain yield under drought stress	5.97
Os07g0541500	Similar to KI domain interacting kinase 1.	*CRK10*	Immune response	4.01
Os10g0101200	Peptidase S10 serine carboxypeptidase family protein.	*OsSCP46*	Grain filling, seed germination	3.97
Os01g0106900	Similar to 1-deoxy-D-xylulose 5-phosphate reductoisomerase.	*OsDXR*	MEP pathway	3.04
Os03g0284100	Similar to two-component response regulator-like PRR73.	*OsCCT11*	Heading date	3.01
Os04g0517100	SG2-type MYB transcription factor.	*OsMYB4*	Biotic stress, drought and cold tolerance	2.82
Os10g0409400	Beta subunit of polygalacturonase 1.	*OsBURP16*	Abiotic stress, cell wall	2.81
Os05g0592800	Splicing variant of protein phosphatase 2C 53	*OsABIL2*	Drought stress, ABA signaling, root architecture	2.57
Os03g0225900	Allene oxide synthase (CYP74A2).	*OsAOS2*	Jasmonic acid synthesis	2.35
Os11g0707000	Similar to ribulose bisphosphate carboxylase/oxygenase activase.	*OsrcaA1, OsrcaA2*	Plant growth, calcium signaling	2.26
Os03g0161800	Similar to SIPL.	*OsARD, OsARD2*	Abiotic stress, submergence	2.26
Os08g0101000	ABI3/VP1 transcription factor family protein.	*IDEF1*	Iron deficiency response	1.48
Os05g0343400	WRKY transcription factor.	*OsWRKY53*	Defense response, wounding response	1.15
Os02g0802500	Similar to H^+-translocating inorganic pyrophosphatase beta-1 polypeptide.	*OVP3*	Anoxia inducible	1.09
Os06g0257450	Ribonucleotide reductase.	*RNRS1, St1,SDL*	Chlorophyll synthesis, plant growth	1.02
Os07g0650600	Similar to BLE2 protein.	*OsBLE2*	Plant growth	-1.01
Os07g0558500	Inositol phosphatase-like protein.	*NYC4*	Leaf senescence	-1.15
Os04g0458200	Similar to ORW1943Ba0077G13.1 protein.	*IIP4*	Secondary wall synthesis	-1.78
Os03g0220100	Similar to very-long-chain fatty acid condensing enzyme	*WSL4*	Leaf cuticular wax synthesis	-1.83

	CUT1.			
Os03g0793000	Zinc finger AN1-type domain containing protein.	*OsiSAP7*	ABA signaling, water-deficit stress	-1.83
Os02g0120800	Similar to small GTP-binding protein.	*OsRacB*	Basal disease resistance, salt tolerance	-1.87
Os01g0662600	Similar to NifU-like protein.	*OsIsu1*	Mitochondrial iron-sulfur-cluster biogenesis	-1.95
Os06g0701700	Ion transporter Na^+/K^+ symport	*OsHKT1, OsHKT2*	Salt tolerance, growth	-2.08
Os05g0411300	Similar to DNA binding protein.	*OsbZIP39*	Endoplasmic reticulum stress response	-3.11
Os04g0401000	Proline-rich protein.	*Pi21*	Disease resistance	-3.45
Os02g0203700	Similar to testis expressed sequence 13A protein.	*SRZ1*	Abiotic stress	-3.91
Os04g0620700	Nucleotide-binding alpha-beta plait domain containing protein.	*OsNUC1*	Salt stress	-4.42
Os08g0237000	Xyloglucan endotransglycosylase/hydrolase protein 8 precursor (EC 2.4.1.207).	*OsXTH8*	Plant growth	-4.80

Only functionally characterized genes are shown. The gene used to test whether its promoter interacts with MoHTR1 was noted (shaded).
*Expression is calculated by $\log_2(FPKM_{36hpi}+1)/(FPKM_{control}+1)$.

Supplementary Table 4. Candidate target genes for MoHTR2.

Locus number	Annotation	Gene name	Function	Expression*
Os02g0121300	Cyclophilin peptidyl-prolyl cis-trans isomerase.	*OsCYP2, OsCyp2-P, LRT2*	Lateral root development, salt tolerance	8.10
Os09g0286600	Pathogenesis-related transcriptional factor and ERF domain containing protein.	*Sub1C*	Submergence tolerance	5.14
Os06g0154200	F-box component of the SKP-Cullin-F box (SCF) E3 ubiquitin ligase complex.	*D3*	Tillering	4.53
Os03g0240600	Zinc finger RING/FYVE/PHD-type domain containing protein.	*OsUPS*	Phosphate starvation	4.40
Os05g0399300	Similar to chitinase.	*Cht-2*	Intracellular chitinase	4.17
Os06g0561000	Similar to myo-inositol oxygenase.	*OsMIOX*	Drought tolerance	4.15
Os02g0218700	Allene oxide synthase (CYP74A3), fatty acid 9-/13-hydroperoxide.	*OsAOS3, OsHPL2*	Bacterial blight resistance	3.41
Os03g0284100	Similar to two-component response regulator-like PRR73.	*OsCCT11*	Heading date	3.01
Os11g0484500	Similar to 6-phosphogluconate dehydrogenase.	*Os6PGDH2*	Abiotic stress responsive	2.52
Os03g0575200	Similar to potassium transporter 1.	*OsHAK16*	Potassium homeostasis, salt tolerance	2.35
Os07g0637300	Similar to pyruvate dehydrogenase kinase.	*OsPDK1*	Gibberellin-mediated growth	2.27
Os01g0802100	Similar to 4-diphosphocytidyl-2-C-methyl-D-erythritol kinase (EC 2.7.1.148).	*OsCMK*	MEP pathway for phytoalexins and isoprenoids	2.12
Os01g0246400	Similar to low molecular mass early light-inducible protein HV90.	*ELIP*	Abiotic stress responsive	2.12
Os06g0354700	Alpha/beta hydrolase-fold family protein.	*NYC3*	Leaf senescence	2.11
Os10g0456800	CHY zinc finger protein.	*DCA1*	Drought and salt tolerance	2.04
Os05g0103200	Cyclophilin-like domain containing protein.	*OsCYP20-2*	Stress tolerance	1.93
Os01g0609300	Similar to pleiotropic drug resistance protein 3.	*Ospdr9*	Hypoxic and salt stress responsive, JA auxin cytokinin responsive	1.81
Os02g0115700	Catalase isozyme A.	*OsCATA*	Water stress responsive	1.75
Os01g0741900	Auxin-responsive protein.	*OsIAA6*	Drought stress	1.68
Os12g0571000	Metallothionein-like protein type 1.	*OsMT1g*	Abiotic stress tolerance	1.63
Os03g0267000	Low molecular mass heat shock protein.	*Oshsp18.0*	Biotic/abiotic stress tolerance	1.58
Os04g0620000	C-type ATP-binding cassette (ABC) transporter.	*OsABCC1, MRP1*	Arsenic accumulation	1.53
Os03g0232600	U-Box E3 ubiquitin ligase.	*TUD1, DSG1, ELF1*	Brassinosteroid-mediated growth,	1.52

			jasmonic acid response	
Os07g0541400	Similar to receptor protein kinase.	*CRK6*	Immune response	1.46
Os05g0460000	Similar to 70 kDa heat shock cognate protein 1.	*OsctHSP70-1*	Heat stress	1.44
Os05g0539800	Protein of unknown function DUF778 family protein.	*OsRTH2*	Ethylene-mediated seddling growth	1.40
Os01g0730300	Similar to predicted protein.	*OsTPS3*	Sesquiterpene synthesis	1.34
Os08g0546800	Similar to heat stress transcription factor B-2b.	*OsHsfB2b*	Drought and salt stress	1.26
Os03g0146400	Similar to lethal leaf-spot 1.	*OsPAO*	Leaf senescence	1.25
Os06g0699400	MAP kinase 2.	*OsMAPK4, OsMSRMK3*	Abiotic stress, development	1.19
Os05g0322900	WRKY transcription factor.	*OsWRKY45*	Salicylic acid signaling, disease resistance	1.09
Os07g0150700	Serine/threonine protein kinase.	*OsCIPK23*	Drought tolerance, pollination	1.02
Os05g0207500	GSK3/SHAGGY-like kinase.	*GSK2*	Brassinosteroid signaling	1.01
Os06g0603600	SPX domain-containing protein.	*OsSPX1*	Phosphate homeostasis, negative regulation of leaf inclination	-1.04
Os03g0230300	Homologue of SRO.	*OsSRO1c*	Regulation of stomatal closure, abiotic stress response	-1.05
Os03g0667100	Similar to NPR1-like protein.	*NH3*	Growth	-1.07
Os05g0474400	Prenylated rab acceptor PRA1 family protein.	*OsPRA1*	Vacuolar trafficking	-1.14
Os06g0644200	Similar to pyrophosphate-energized vacuolar membrane proton pump.	*OVP1*	Cold tolerance	-1.15
Os04g0194600	Similar to PCF1.	*PCF1*	Meristem	-1.18
Os03g0717000	Similar to TMK protein precursor.	*OsTMK*	Plant growth	-1.22
Os07g0515100	Calcium-dependent protein kinase isoform 2 (EC 2.7.1.-).	*OsCDPK2, OsCPK2*	Seed development	-1.26
Os02g0668100	Similar to geranylgeranyl-diphosphate synthase.	*OsGGPPS2*	Chlorophyll biosynthesis	-1.29
Os06g0683400	Small calcium-binding protein with one EF-hand motif.	*CCD1*	Carotenoid cleavage	-1.30
Os05g0530500	Similar to OSK1.	*SnRK1A*	Seed germination	-1.33
Os01g0667900	CC-type glutaredoxin.	*OsGRX6*	Senescence, nitrogen homeostasis	-1.36
Os05g0472700	Similar to metal transport protein.	*OsZIP5*	Zinc transporter	-1.36
Os04g0556300	Glutathione peroxidase.	*OsGPX1*	Photosynthesis, salinity response	-1.37
Os04g0541700	Similar to homeobox-leucine zipper protein HOX22.	*Oshox22*	ABA-mediated drought and salt tolerance	-1.52
Os03g0815100	Similar to OsNAC6 protein.	*OsNAC19,*	Drought tolerance	-1.55

		SNAC1, OsNAC9		
Os06g0131700	Similar to NAM protein.	*OsSWN1*	Lignin biosynthetic gene expression	-1.79
Os07g0695100	Pseudo response regulator.	*Ghd7.1, Hd2, OsPRR37, DTH7*	Grain yield	-1.80
Os04g0653000	Tify domain containing protein.	*OsJAZ1, OsTIFY3, EG2*	Drought tolerance	-1.82
Os03g0576900	Amino acid/polyamine transporter I family protein.	*OsPAR1*	Intracellular paraquat transport	-1.82
Os06g0600400	Cytochrome P450 family protein.	*CYP734A4*	Brassinosteroid catabolism	-1.86
Os02g0120800	Similar to Small GTP-binding protein.	*OsRacB*	Basal disease resistance, salt tolerance	-1.87
Os01g0662600	Similar to NifU-like protein.	*OsIsu1*	Mitochondrial iron-sulfur-cluster biogenesis	-1.95
Os11g0499600	NAD(P)-binding domain containing protein.	*OsHSD1, LGF1*	Cuticle formation	-1.95
Os02g0618200	Signal transduction response regulator receiver region domain containing protein.	*OsPRR1*	Flowering time	-2.01
Os10g0494300	ATP binding cassette G transporter.	*OsABCG26*	Regulation of male reproduction, anther cuticle development	-2.07
Os05g0381400	AWPM-19-like protein.	*OsPM19L1*	Stress tolerance	-2.18
Os02g0745100	Aquaporin NIP III subfamily protein.	*Lsi1, OsNIP2;1, OsLsi1*	Silicon/arsenite transport	-2.30
Os04g0660400	Similar to amylogenin.	*OsUAM2*	Cell wall	-2.36
Os10g0405500	Coiled-coil protein.	*PAIR3*	Meiosis	-2.39
Os05g0386800	COBRA-like protein.	*OsBC1L4*	Cell wall	-2.44
Os12g0460800	Similar to lammer-type protein kinase.	*OsDR11*	Disease resistance	-2.52
Os07g0545800	Similar to chitin-inducible gibberellin-responsive protein.	*CIGR1*	Chitin responsive	-2.58
Os11g0523800	Similar to Isoform 3 of auxin response factor 23.	*OsARF1*	Ausin response, seed development, growth	-2.69
Os04g0641700	Similar to H0423H10.4 protein.	*ILI1*	Brassinosteroid response, growth	-2.73
Os09g0540500	Mediator complex subunit Med4 domain containing protein.	*OsMED4*	Plant development	-2.78
Os07g0622000	Serine/threonine protein kinase.	*SAPK2*	Hyperosmotic stress response, Abscisic acid-dependent gene regulation	-2.89
Os07g0596300	Actin-binding FH2 domain containing protein.	*FH5, RMD, BUI1*	Plant growth, development	-2.96
Os03g0214200	Protein of unknown function DUF1675 family protein.	*MODD*	Drought tolerance	-3.17

Os03g0820500	Similar to WCOR719.	*OsADF3*	Drought tolerance	-3.53
Os02g0724000	CONSTANS-like protein.	*DTH2, OsCOL9, OsCCT08*	Flowering time, disease resistanse	-3.67
Os01g0968800	Similar to dehydration responsive element binding protein 1F.	*OsDREB1F, RCBF2*	Salt/drought/cold tolerance	-4.31
Os01g0971800	Similar to two-component response regulator ARR11 (Receiver-like protein 3).	*OsPCL1*	Circadian rhythm	-4.58
Os07g0154100	Similar to viviparous-14.	*OsNCED4*	Drought/salt tolerance, ABA synthesis	-4.71
Os06g0597500	Pentatricopeptide repeat domain containing protein.	*sped1-D*	Inflorescence branch development	-4.83
Os05g0111300	Similar to B22EL8 protein.	*OsMT2b*	Root development, seed germination, disease susceptibility	-4.88
Os04g0531100	C2 calcium-dependent membrane targeting domain containing protein.	*Rpp16, OsPBP1*	Pollen fertility	-8.60

Only functionally characterized genes are shown. Two genes used to test whether their promoters interact with MoHTR2 were noted (shaded).

*Expression is calculated by $\log_2(FPKM_{36hpi}+1)/(FPKM_{control}+1)$.

Supplementary Table 5. Candidate genes targeted by both MoHTR1 and MoHTR2.

Locus number	Annotation	Gene name	Function	Expression*
Os09g0572000	Pathogenesis-related transcriptional factor and ERF domain containing protein.	-	-	4.65
Os03g0284100	Similar to Two-component response regulator-like PRR73.	*OsCCT11*	-	2.85
Os05g0101400	Similar to 50S ribosomal protein L28 chloroplast precursor.	-	-	1.69
Os03g0278566	Conserved hypothetical protein.	-	-	1.54
Os12g0612300	Conserved hypothetical protein.	-	-	1.22
Os08g0158900	GTP1/OBG domain containing protein.	-	-	-1.02
Os06g0232000	Similar to Pro-resilin.	-		-1.05
Os02g0120800	Similar to Small GTP-binding protein.	*OsRacB*	salicylic acid, jasmonic acid, disease resistance, salt tolerance, development	-1.80
Os10g0391400	Tify domain containing protein.	-	-	-3.32
Os06g0219900	Similar to Phi-1 protein.	-	-	-4.24
Os07g0208533	Hypothetical gene.	-	-	-7.58

*Expression is calculated by $\log_2(FPKM_{36hpi}+1)/(FPKM_{control}+1)$.

Supplementary Table 6. GO enrichment analysis of the candidate genes targeted by MoHTR1.

GO term	Description	*P*-value	Number of genes
GO:0007582	Physiological process	9.70E-20	124
GO:0009987	Cellular process	1.34E-14	103
GO:0008152	Metabolism	3.49E-14	104
GO:0006950	Response to stress	3.16E-09	40
GO:0050896	Response to stimulus	1.17E-08	49
GO:0050875	Cellular physiological process	1.91E-08	77
GO:0044238	Primary metabolism	1.77E-04	62
GO:0044237	Cellular metabolism	3.29E-04	51
GO:0009719	Response to endogenous stimulus	5.80E-04	18
GO:0009628	Response to abiotic stimulus	1.43E-03	22
GO:0051234	Transport	1.63E-03	23
GO:0051179	Localization	1.63E-03	23
GO:0006810	Establishment of localization	1.63E-03	23

Supplementary Table 7. GO enrichment analysis of the candidate genes targeted by MoHTR2.

GO term	Description	*P*-value	Number of genes
GO:0007582	Physiological process	6.88E-44	337
GO:0009987	Cellular process	2.42E-36	287
GO:0008152	Metabolism	8.71E-35	289
GO:0050875	Cellular physiological process	4.09E-24	222
GO:0044238	Primary metabolism	1.44E-21	202
GO:0044237	Cellular metabolism	7.94E-18	162
GO:0050896	Response to stimulus	3.55E-17	125
GO:0043170	Macromolecule metabolism	5.82E-12	122
GO:0009058	Biosynthesis	1.05E-11	116
GO:0009719	Response to endogenous stimulus	3.14E-10	49
GO:0019538	Protein metabolism	2.60E-09	96
GO:0009628	Response to abiotic stress	4.52E-09	60
GO:0007154	Cell communication	2.56E-08	51
GO:0043283	Biopolymer metabolism	4.35E-08	75
GO:0044260	Cellular macromolecule metabolism	1.02E-07	78
GO:0044267	Cellular protein metabolism	1.02E-07	78
GO:0007165	Signal transduction	1.33E-07	43
GO:0006950	Response to stress	2.14E-07	75
GO:0006139	Nucleobase, nucleoside, nucleotide and nucleic acid metabolism	2.23E-07	83
GO:0006464	Protein modification	6.52E-07	66
GO:0043412	Biopolymer modification	6.52E-07	66
GO:0006810	Transport	1.03E-04	52
GO:0051179	Localization	1.03E-04	52
GO:0051234	Establishment of localization	1.03E-04	52
GO:0016043	Cell organization and biogenesis	2.12E-03	34
GO:0007275	Development	6.78E-03	52

Supplementary Table 8. GO enrichment analysis of the differentially expressed genes in *MoHTR1*-OX.

Upregulated DEGs

GO term	Description	*P*-value	Number of genes
GO:0007582	physiological process	5.89E-27	196
GO:0008152	metabolism	7.40E-24	173
GO:0009987	cellular process	1.02E-20	164
GO:0050896	response to stimulus	5.63E-17	86
GO:0006950	response to stress	1.26E-14	65
GO:0044238	primary metabolism	3.21E-14	120
GO:0050875	cellular physiological process	2.30E-13	126
GO:0009628	response to abiotic stress	7.84E-12	47
GO:0044237	cellular metabolism	4.01E-11	95
GO:0009058	biosynthesis	1.33E-10	76
GO:0006139	nucleobase, nucleoside, nucleotide and nucleic acid metabolism	7.37E-08	57
GO:0009719	response to endogenous stimulus	2.05E-07	31
GO:0007275	development	0.000107	39
GO:0043170	macromolecule metabolism	0.00531	58

Downregulated DEGs

GO term	Description	*P*-value	Number of genes
GO:0007582	physiological process	3.25E-75	459
GO:0009987	cellular process	6.64E-55	658
GO:0008152	metabolism	5.46E-53	383
GO:0050875	cellular physiological process	1.31E-30	281
GO:0044238	primary metabolism	9.15E-29	259
GO:0050896	response to stimulus	2.67E-26	168
GO:0006950	response to stress	1.73E-19	119
GO:0043170	macromolecule metabolism	1.07E-17	161
GO:0044237	cellular metabolism	7.70E-15	184
GO:0009058	biosynthesis	1.94E-12	140
GO:0006810	transport	1.42E-11	80
GO:0051179	localization	1.42E-11	80
GO:0051234	establishment of localization	1.42E-11	80
GO:0019538	protein metabolism	2.60E-11	120
GO:0009628	response to abiotic stimulus	3.93E-11	75
GO:0009719	response to endogenous stimulus	1.83E-09	55
GO:0009056	catabolism	1.39E-08	53
GO:0009607	response to biotic stimulus	9.98E-08	41
GO:0006139	nucleobase, nucleoside, nucleotide and nucleic acid metabolism	7.94E-07	97

GO:0044260	cellular macromolecule metabolism	1.07E-05	86
GO:0044267	cellular protein metabolism	1.07E-05	86
GO:0043283	biopolymer metabolism	1.29E-05	81
GO:0006464	protein modification	1.40E-05	74
GO:0043412	biopolymer modification	1.40E-05	74
GO:0005975	carbohydrate metabolism	1.11E-04	35
GO:0007154	cell communication	2.58E-04	49
GO:0015979	photosynthesis	3.87E-04	14
GO:0007275	development	2.87E-03	64
GO:0007165	signal transduction	5.70E-03	38

Supplementary Table 9. GO enrichment analysis of the differentially expressed genes in *MoHTR2*-OX

Upregulated DEGs

GO term	Description	*P*-value	Number of genes
GO:0007582	physiological process	5.35E-29	180
GO:0008152	metabolism	2.01E-28	164
GO:0050896	response to stimulus	1.94E-19	83
GO:0009987	cellular process	4.71E-17	141
GO:0006950	response to stress	1.44E-16	63
GO:0009628	response to abiotic stimulus	1.58E-15	49
GO:0044238	primary metabolism	8.06E-11	101
GO:0009058	biosynthesis	1.77E-10	69
GO:0009719	response to endogenous stimulus	3.03E-08	30
GO:0050875	cellular physiological process	1.88E-06	96
GO:0044237	cellular metabolism	2.91E-06	74
GO:0019748	secondary metabolism	5.79E-06	14
GO:0009607	response to biotic stimulus	1.26E-05	21
GO:0006139	nucleobase, nucleoside, nucleotide and nucleic acid metabolism	7.00E-05	45
GO:0007275	development	6.67E-03	31
GO:0009056	catabolism	8.53E-03	20

Downregulated DEGs

GO term	Description	*P*-value	Number of genes
GO:0007582	physiological process	2.58E-67	448
GO:0008152	metabolism	1.11E-49	378
GO:0009987	cellular process	6.60E-44	360
GO:0044238	primary metabolism	2.68E-32	268
GO:0050875	cellular physiological process	8.38E-24	265
GO:0050896	response to stimulus	9.51E-24	163
GO:0006950	response to stress	2.13E-18	117
GO:0009058	biosynthesis	2.06E-14	146
GO:0044237	cellular metabolism	2.19E-12	177
GO:0043170	macromolecule metabolism	4.28E-12	146
GO:0009628	response to abiotic stimulus	6.12E-12	77
GO:0009719	response to endogenous stimulus	1.82E-08	53
GO:0006629	lipid metabolism	1.84E-08	42
GO:0006139	nucleobase, nucleoside, nucleotide and nucleic acid metabolism	1.18E-07	100
GO:0019538	protein metabolism	4.99E-07	107
GO:0009056	catabolism	6.27E-06	47
GO:0006810	transport	1.05E-05	65

GO:0051179	localization	1.05E-05	65
GO:0051234	establishment of localization	1.05E-05	65
GO:0005975	carbohydrate metabolism	1.63E-05	37
GO:0015979	photosynthesis	7.97E-05	15
GO:0009607	response to biotic stimulus	4.81E-04	33
GO:0043283	biopolymer metabolism	2.19E-03	73
GO:0006464	protein modification	2.85E-03	66
GO:0043412	biopolymer modification	2.85E-03	66
GO:0007275	development	3.18E-03	64
GO:0044260	cellular macromolecule metabolism	4.88E-03	76
GO:0044267	cellular protein metabolism	4.88E-03	76

Supplementary Table 10. Growth and developmental phenotypes of the *MoHTR* knockout mutants.

Strain	Vegetative growth[a] (mm)		Conidiation[b] ($\times 10^4$/mL)	Germination[c] (%)	Appressoria formation[d] (%)
	CM	MM			
Wild type	66.8 ± 0.3	65.3 ± 0.6	48.7 ± 1.5	94.3 ± 2.5	96.7 ± 0.6
Δ*Mohtr1*	66.7 ± 0.6	64.8 ± 0.3	51.1 ± 2.0	93.7 ± 1.5	96.0 ± 1.0
Δ*Mohtr2*	66.8 ± 0.3	65.2 ± 1.3	47.9 ± 5.0	95.0 ± 2.6	97.7 ± 0.6

[a]Colony diameters on complete medium (CM) and minimal medium (MM) were measured at 9 days post inoculation (dpi).
[b]Conidia were collected from cultures on V8 agar at 6 dpi and counted.
[c]Percentages of germinated conidia on hydrophobic surface at 12 hpi.
[d]Percentages of the conidia that formed the appressorium on hydrophobic at 12 hpi.

Supplementary Table 11. Primers used in this study.

Name	Sequence
**Primers for protein localization**	
attB_F1	GGGGACAAGTTTGTACAAAAAAGCAGGCT
attB_R1	GGGGACCACTTTGTACAAGAAAGCTGGGT
MoHTR1_attB_F	AAAAAGCAGGCTTACATCAGTGGGACATGGGCTT
MoHTR1_attB_R	AGAAAGCTGGGTAGAGCTGGACCGGGTCG
MoHTR2_attB_F	AAAAAGCAGGCTTAGCACCGAGTAGGCACAGTG
MoHTR2_attB_R	AGAAAGCTGGGTATGTGGGTCGATTCCAGCT
MoHTR1_F w/ PEF1a	TCACAAAAGGAACCCAATCTTCAAAATGCAGCTTTCAAACTTTCTT
MoHTR1_F w/ EF1a w/o sp	TCACAAAAGGAACCCAATCTTCAAAATGGCCCCCATGCCTTCCGG
MoHTR1_R w/o stop	TTGCTCACCATACCACCACCGAGCTGGACCGGGTCGC
MoHTR2_F w/ PEF1a	TCACAAAAGGAACCCAATCTTCAAAATGCATCTCAAAGCCTCCAG
MoHTR2_F w/ EF1a w/o sp	TCACAAAAGGAACCCAATCTTCAAAATGTACCCAGCGTCGGCCAAC
MoHTR2_R w/o stop	TTGCTCACCATACCACCACCTGTGGGTCGATTCCAGCTATGT
EF1a_attB_F	AAAAAGCAGGCTTACGGTACCTATAGGGCGAATTG
EF1a_R	TTTGAAGATTGGGTTCCTTTTGTGA
**Primers for knockout mutant generation**	
MoHTR1_UF	TTCATCAGTGGGACATGGGC
MoHTR1_UR	CCTCCACTAGCTCCAGCCAAGCCTGGCAGAGGTATGTCACGAG
MoHTR1_DF	GTTGGTGTCGATGTCAGCTCCGGAGACACACATTACAGGCTGGGC
MoHTR1_DR	AGCGATACGACGCCACCATC
MoHTR2_UF	ACAGCAACCCAGTAGCTTTG
MoHTR2_UR	CCTCCACTAGCTCCAGCCAAGCCTTGACACGAAAGAAACGGGA
MoHTR2_DF	GTTGGTGTCGATGTCAGCTCCGGAGCAGAGATTGAATGCGGGTACG
MoHTR2_DR	CGCAATTGGGCGTTAAATACCC
HYR_F1	GGCTTGGCTGGAGCTAGTGGAGG
HYR_R1	CTCCGGAGCTGACATCGACACCAAC
**Primers for quantitative RT-PCR**	
Rice act_RT_F	GCGTGGACAAAGTTTTCAACCG
Rice act_RT_R	TCTGGTACCCTCATCAGGCATC
MoHTR1_RT_F	GCCCTGGAATCAATACGGAG
MoHTR1_RT_R	GAGTCTATCGTTGCCAGGAAG
MoHTR2_RT_F	GTGCGGTTATTGCGGTAAAAG
MoHTR2_RT_R	GCTATGTTCTTTGTAGGGCTGG
MYB4_RT_F	TCTGAATTCTGTGCTACGCAG

MYB4_RT_R	TTCTTGATCTCGTTGTCCGTC
HPL2_RT_F	CTACTCCTGCACACCTTCTC
HPL2_RT_R	AGGAAGATCTTGAAGCCGC
WRKY45_RT_F	AGCAATCGTCCGGGAATTC
WRKY45_RT_R	CTTTGGGTGCTTGGAGTTTTG
PR1a_RT_F	GGCACGAGTCGATCTCCA
PR1a_RT_R	ACCAGCAAGCAGCAGGAT
PR1b_RT_F	GGCAACTTCGTCGGACAGA
PR1b_RT_R	CCGTGGACCTGTTTACATTTT
PR2_RT_F	ACATCAAGGTGACGACGTCG
PR2_RT_R	ATCAGAAGCTGATGGGGTAG
PR3_RT_F	GGCGTTCTGGTTCTGGATGAC
PR3_RT_R	CGCCGTTGATGATGTTGGTC
PR4_RT_F	TGGGACCTGAACAAAGTGAGC
PR4_RT_R	TGGATACACTTGCCACACGAG
PR5_RT_F	AACTACCAGGTCGTCTTCTGC
PR5_RT_R	GCGTGCGGGCTTATTCTTA
PR10a_RT_F	ACACTCGACGGAGACGAAGC
PR10a_RT_R	CAGGGTGAGCGACGAGGTA
POX22.3_RT_F	GGCAAATACCGACCTCCCT
POX22.3_RT_R	TCGTTGTAGATCCTGTCCCTGA
PAL_RT_F	CCCTGCCAATCTGCTGAACTA
PAL_RT_R	GCCGCTATGCAACGAAGAAT
LOX2_RT_F	CGATGGCCGGAACAAGGATA
LOX2_RT_R	TGGAGCGTTTTGTCTCATCA
NPR1_RT_F	CACGCCTAAGCCTCGGATTA
NPR1_RT_R	TCAGTGAGCAGCATCCTGACTAG
JAMYB_RT_F	TGGCGAAACGATGGAGATGG
JAMYB_RT_R	CCTCGCCGTGATCAGAGATG
JiOsPR10_RT_F	CGGACGCTTACAACTAAATCG
JiOsPR10_RT_R	AAACAAAACCATTCTCCGACAG
EBP89_RT_F	TGACGATCTTGCTGAACTGAA
EBP89_RT_R	CAATCCCACAAACTTTACACA
ACS1_RT_F	TCGGCCAAGACCCTCGACG
ACS1_RT_R	CGAAAGGAATCTGCTACTGCTGC
**Primers for yeast one hybrid**	
MoHTR1_w/ stop_DTOPO_R	TCAGAGCTGGACCGGGTCGC
MoHTR2_w/ stop_DTOPO_R	TCATGTGGGTCGATTCCAGCTATGT

OsHPL2_pro1_EcoI_F	AATTATTATATTTTGAAATGGAGGTGGTATTTAATTTATAAG
OsHPL2_pro1_SalI_R	TCGACTTATAAATTAAATACCACCTCCATTTCAAAATATAAT
OsHPL2_pro2_EcoI_F	AATTCAGCTCATTTTAAATGGAGGTGGATATATTTTTCACCC
OsHPL2_pro2_SalI_R	TCGAGGGTGAAAAATATATCCACCTCCATTTAAAATGAGCTG
WRKY45_pro_EcoRI_ F	AATTCACACCACCACACCACCACCTCCTCATCCTCATTTGCC
WRKY45_pro_SalI_R	TCGAGGCAAATGAGGATGAGGAGGTGGTGGTGTGGTGGTGTG
OsMYB4_pro_EcoRI_ F	AATTTGATGGAAAATGACTGAAGATTGGATCTAAACTTTGTA
OsMYB4_pro_SalI_R	TCGATACAAAGTTTAGATCCAATCTTCAGTCATTTTCCATCA
**Primers for transgenic rice generation**	
MoHTR1_w/o sp_DTOPO_F	CACCATGGCCCCCATGCCTTCCGG
MoHTR1_w/o stop_DTOPO_R	GAGCTGGACCGGGTCGC
MoHTR2_w/o sp_DTOPO_F	CACCATGTACCCAGCGTCGGCCAAC
MoHTR2_w/o stop_DTOPO_R	TGTGGGTCGATTCCAGCTATGT
