# Supplementary material for: Two nuclear effectors of the rice blast fungus modulate host immunity via transcriptional reprogramming
Source: Nat Commun. 2020 Nov 17;11:5845. doi: 10.1038/s41467-020-19624-w (PMC7672089; doi:10.1038/s41467-020-19624-w)
Supplement: Supplementary file 3 — Reporting Summary [file 41467_2020_19624_MOESM3_ESM.pdf]

nature research

Corresponding author(s): Yong-Hwan Lee

Last updated by author(s): Oct 12, 2020

Reporting Summary

Nature Research wishes to improve the reproducibility of the work that we publish. This form provides structure for consistency and transparency in reporting. For further information on Nature Research policies, see Authors & Referees and the Editorial Policy Checklist .

Statistics

For all statistical analyses, confirm that the following items are present in the figure legend, table legend, main text, or Methods section.

n/a	Confirmed	
☐	☒	The exact sample size (*n*) for each experimental group/condition, given as a discrete number and unit of measurement
☐	☒	A statement on whether measurements were taken from distinct samples or whether the same sample was measured repeatedly
☐	☒	The statistical test(s) used AND whether they are one- or two-sided<br>*Only common tests should be described solely by name; describe more complex techniques in the Methods section.*
☒	☐	A description of all covariates tested
☐	☒	A description of any assumptions or corrections, such as tests of normality and adjustment for multiple comparisons
☐	☒	A full description of the statistical parameters including central tendency (e.g. means) or other basic estimates (e.g. regression coefficient) AND variation (e.g. standard deviation) or associated estimates of uncertainty (e.g. confidence intervals)
☐	☒	For null hypothesis testing, the test statistic (e.g. *F*, *t*, *r*) with confidence intervals, effect sizes, degrees of freedom and *P* value noted<br>*Give P values as exact values whenever suitable.*
☒	☐	For Bayesian analysis, information on the choice of priors and Markov chain Monte Carlo settings
☒	☐	For hierarchical and complex designs, identification of the appropriate level for tests and full reporting of outcomes
☒	☐	Estimates of effect sizes (e.g. Cohen's *d*, Pearson's *r*), indicating how they were calculated

Our web collection on statistics for biologists contains articles on many of the points above.

Software and code

Policy information about availability of computer code

Data collection	Zeiss ZEN was use for confocal microscopy; AxioVison 4.8 was used for epifluorescence microscopy; GenePix® Pro Microarray Image Analysis Software was used for protein binding microarray; Rotor-Gene Q Software was used for quantitative RT-PCR; Wallac 1420 was used to quantify luminescence; ImageJ was used for measurement of lesion size.
Data analysis	IBM SPSS Statistics 25 was used for determining the significance of the data.

For manuscripts utilizing custom algorithms or software that are central to the research but not yet described in published literature, software must be made available to editors/reviewers. We strongly encourage code deposition in a community repository (e.g. GitHub). See the Nature Research guidelines for submitting code & software for further information.

Data

Policy information about availability of data

All manuscripts must include a data availability statement. This statement should provide the following information, where applicable:

- Accession codes, unique identifiers, or web links for publicly available datasets
- A list of figures that have associated raw data
- A description of any restrictions on data availability

All data that support the findings of this study are present in the paper, Supplementary information, and Source data. RNA-seq data has been deposited into the NCBI Sequence Read Archive under accession number PRJNA668035 [https://www.ncbi.nlm.nih.gov/bioproject/ PRJNA668035].

Field-specific reporting

Please select the one below that is the best fit for your research. If you are not sure, read the appropriate sections before making your selection.

- [x] Life sciences
- [ ] Behavioural & social sciences
- [ ] Ecological, evolutionary & environmental sciences

For a reference copy of the document with all sections, see nature.com/documents/nr-reporting-summary-flat.pdf

Life sciences study design

All studies must disclose on these points even when the disclosure is negative.

Sample size	The sample sizes were decided following the previous studies. Experiments were carried out with as many samples as possible and sufficient to obtain reproducible results. Exact sample size was noted in the manuscript.
Data exclusions	No data were excluded.
Replication	The experiments were repeated at least three times independently with similar results. Exact replication numbers were noted in the manuscript.
Randomization	Randomly selected fungal colonies and rice plants seedlings were used for comparing different phenotypes. Position of sample tubes in the experimental instrument was randomized for qRT-PCR and transcription activity assay.
Blinding	Main results were observed by two investigators. Fungal virulence, rice susceptibility, qRT-PCR, and transcription activity assay were conducted with the genotype unidentifiable label.

Reporting for specific materials, systems and methods

We require information from authors about some types of materials, experimental systems and methods used in many studies. Here, indicate whether each material, system or method listed is relevant to your study. If you are not sure if a list item applies to your research, read the appropriate section before selecting a response.

Materials & experimental systems

n/a	Involved in the study
[x]	[ ] Antibodies
[x]	[ ] Eukaryotic cell lines
[x]	[ ] Palaeontology
[x]	[ ] Animals and other organisms
[x]	[ ] Human research participants
[x]	[ ] Clinical data

Methods

n/a	Involved in the study
[x]	[ ] ChIP-seq
[x]	[ ] Flow cytometry
[x]	[ ] MRI-based neuroimaging
