# Supplementary material for: Two nuclear effectors of the rice blast fungus modulate host immunity via transcriptional reprogramming
Source: Nat Commun. 2020 Nov 17;11:5845. doi: 10.1038/s41467-020-19624-w (PMC7672089; doi:10.1038/s41467-020-19624-w)
Supplement: Supplementary file 2 — Peer Review File [file 41467_2020_19624_MOESM2_ESM.pdf]

Reviewer #1 (Remarks to the Author):

In this study, the authors identified and characterized two putative effector genes, MoHTR1 and MoHTR2, in Magnaporthe oryzae. Both of them are expressed during plant infection and have zinc finger domains. During infection, they were secreted by BIC and enriched in plant nuclei. The authors then identified putative binding sequences of Htr1/Htr2 with a 9-mer-based protein binding microarray, verified their binding by yeast one-hybrid assays, and used them to search for candidate target genes of Htr1/Htr2 in rice by analyzing the promoters of rice genes with altered expression levels during M. oryzae infection. Transient expression of Htr1 and Htr3 suppressed the expression of LUC under the control of OsMYB4 and OsWRK45 promoters (their corresponding targets). Transgenic rice plants overexpressing HTR1 or HTR2 were increased their susceptibility to M. oryzae and Xoo but increased their resistance against C. myyabeanus.

This is a well-written manuscript. Although many effector proteins have been characterized in M. oryzae, Htr1 and Htr2 are the first to nuclear effectors characterized in this model fungus.

1. Better characterization and descriptions of Htr1/Htr2 are necessary. For the htr1 and htr2 deletion mutants, what happens to the differentiation and growth of invasive hyphae in rice leaf sheath cells? What about plant defense responses in comparison with the wild type? What about their virulence in infection assays with barley leaves? What are the expression profiles of these two HTR genes in different infection and development stages? Are they specifically expressed in invasive hyphae? Do they have NLS? If so, are their NLS necessary for their functions or localization during plant infection? Also, the rationale for selecting HTR1 and HTR2 (the sentence on lines 119-120) is not clear (I assume there are more than two HTRs fitting these criteria). Why these two HTR genes? Also, why not just select one?
What is the relationship between Htr1 and Htr2? They differed in protein sequences and DNA binding sites, and affected different genes in rice plants. Why did their overexpression have the same effects on resistance/susceptibility to Mo, Xoo, and Cm? Xoo is an apoplastic bacterial pathogen.

2. Because the authors used the putative binding sites of Htr1 and Htr2 to identify their target genes in rice, it will be helpful to use additional approaches other than yeast one-hybrid to confirm these binding sites, such as gel mobility shift assays and site-directed mutagenesis of the promoters of selected target genes (OsMYB4, OSsWRK45?).

3. The authors generated Htr1OE and Htr2OE transgenic rice and conducted RNA-seq analysis with these materials. They also showed that both had negative regulation. What is the percentage of the genes with down-regulated expression levels in Htr1OE or Htr2OE plants have the putative binding sites? What about the genes up-regulated or not affected have the putative binding sites? Any enrichment? How many of the genes affected by Htr1OE have the Htr2 recognition site and vice versa? The authors generated the htr1 and htr2 deletion mutants in M. oryzae and did some qRT-PCR assays. I am not sure whether they have RNA-seq data of rice leaves infected with these two mutants. If they did, it will be interesting to include (some sentences gave me the impression they had RNA-seq data).

4. Another question related to the targets of Htr1 and Htr2. How many of these 226 and 650 genes (line 186) were up-regulated or down-regulated? How many of them were common between Htr1OE and Htr2OE plants? How many of them or their homologs have been shown to be related to plant immunity? The authors described three of them on 193-194. Are these three genes the only ones? Are OsMYB4, OsHPL2, and OsWRKY45 induced and/or activated during resistance against bacterial and/or fungal infections (based on published data)? Expression vs activation. Were their expression levels still affected by M. oryzae infection?
Were the expression of OsHPL2 and OsWRKY45 affected in the htr1OE plants? Were the expression of OsMYB4 affected in the htr2OE plants? (Just curious, are the Htr1OE and Htr2OE plants normal in growth and development? Many rice genes may have the binding sites of Htr1 and Htr2 and their expression levels were higher than actin)

Minor points

1. The title should be more specific. Two nuclear effectors or Nuclear effects Htr1 and Htr2.
2. Is Mo necessary to be included in the gene names here? Will HTR1 and HTR2 be more suitable for these two genes?
3. Line 153, I am not sure whether it has been experimentally proved about moving through plasmodesmata beyond any doubt.
4. What is the average length of 5'UTR for rice? Is 1-kb somewhat too short? (Line 184-186)

Reviewer #2 (Remarks to the Author):

This study functionally characterized two nuclear effectors of M. oryzae. The authors also generated transgenic rice line expressing these effectors that show increased susceptibility to M. oryzae and Xanthomonas oryzae pv. oryzae, hemibiotrophic pathogens, as well as greater resistance to Cochliobolus miyabeanus, a necrotrophic pathogen. in rice virulence of 192 isolates of Magnaporthe oryzae against several rice varieties carrying known resistance genes. The results and conclusions are appropriate. The manuscript is well written, but lacks appropriate review of literature. The outcomes should serve as references for fungal geneticist. I support the publication of this manuscript after proper corrections.

I have some comments that the authors should address. There is one problem in the manuscript:
- Lack of the literature review related to the last 6-7 years. There are a couple of excellent papers on fungal nuclear effectors in "high-profile" journals.

Specific comments:
Abstract, Line 28: "no nuclear effectors have been found in fungal pathogens"; The authors should check the literature" There are nuclear effectors described in other fungal pathogens, such as the See1 nuclear effector of the maize smut fungus, Ustilgo maydis (Redkar et al. 2015. Plant Cell. 27: 1332–1351), the CgEP1, a nuclear effector of the maize pathogen Colletotrichum graminicola (Vargas et al., 2016. MPMI. 29:83-95), and the PstGSRE1, of the wheat stripe rust pathogen, Puccinia striiformis f. sp. tritici (Qi et al., 2019. Molecular Plant 12: 1624-1638).

Introduction, Line 42: "Plants employ multi-layered systems for protection…" It is a two-layered system. The first layer, also called basal defense, involve PAMP and PRR receptors that triggers PAMP-triggered immunity (PTI). The second layer involves AVR-effectors and NBS-LRR receptors that triggers effector-triggered immunity (ETI).

Introduction, Line 56-62: the authors should describe in detail the fungal nuclear effectors of U. maydis, C. graminicola and P. striiformis f. sp. Tritici. These effectors were well functionally characterized and there is a rich literature on the topic.

Results, Line 195: There many assays to determine DNA-protein interaction, all can give false positives. The most commonly used techniques to determine DNA-protein interactions in vitro techniques are footprinting assays, electrophoretic mobility shift assay, southwestern blotting, yeast one-hybrid assay, phage display and proximity ligation assay. The authors should careful conclude their DNA-protein interaction results. Two accurate in vitro methods should be used.

Methods, line 316: Chimeric proteins used in the protein-binding microarray were expressed in E. coli. The authors should use Pichia pastoris or other eukaryotic microorganism for expressing eukaryotic

fungal gene for appropriate post translational modifications of protein maturation between other protein modifications processes specific of eukaryotic organisms.

Response to Referees Comments and Suggestions

Reviewer #1 (Remarks to the Author):

In this study, the authors identified and characterized two putative effector genes, MoHTR1 and MoHTR2, in Magnaporthe oryzae. Both of them are expressed during plant infection and have zinc finger domains. During infection, they were secreted by BIC and enriched in plant nuclei. The authors then identified putative binding sequences of Htr1/Htr2 with a 9-mer-based protein binding microarray, verified their binding by yeast one-hybrid assays, and used them to search for candidate target genes of Htr1/Htr2 in rice by analyzing the promoters of rice genes with altered expression levels during M. oryzae infection. Transient expression of Htr1 and Htr3 suppressed the expression of LUC under the control of OsMYB4 and OsWRK45 promoters (their corresponding targets). Transgenic rice plants overexpressing HTR1 or HTR2 were increased their susceptibility to M. oryzae and Xoo but increased their resistance against C. myyabeanus.

This is a well-written manuscript. Although many effector proteins have been characterized in M. oryzae, Htr1 and Htr2 are the first to nuclear effectors characterized in this model fungus.

1. Better characterization and descriptions of Htr1/Htr2 are necessary.
For the htr1 and htr2 deletion mutants, what happens to the differentiation and growth of invasive hyphae in rice leaf sheath cells?

Δ*Mohtr1* and Δ*Mohtr2* showed significantly reduced invasive growth compared to the wild-type strain (KJ201) when they were inoculated on sheaths of a susceptible rice variety. In plants infected with Δ*Mohtr1* and Δ*Mohtr2*, the numbers of infection sites that showed invasive hyphal growth across multiple rice cells were reduced to 34.0% and 39.4%, respectively, of that observed in plants infected with KJ201 at 48 hours after inoculation. This result supports the significant roles of MoTHR1 and MoTHR2 during the early stage of infection (described in lines 283~289 and shown in Supplementary Fig. 7).

What about plant defense responses in comparison with the wild type?

Infection with each deletion mutant caused increased expression of plant immunity-associated genes, such as pathogenesis-related (*PR*) genes and hormone signaling genes. Some genes, including *PR5*, *PR10a*, and SA signaling-related genes, were commonly induced in plants infected with Δ*Mohtr1* and Δ*Mohtr2*. These are described in lines 258~263 and are presented in Supplementary Fig. 5.

What about their virulence in infection assays with barley leaves?

We very much appreciate this question. We have not yet performed the experiment because we do not believe that it is a crucial experiment to perform. Addressing this question properly requires more than just infecting barley plants with these mutants and should be a focus of our follow-up study for the following reasons. Considering genomic differences between rice and barley, we need to compare sequences of the candidate target

genes between two cereals and analyze barley-mutant interactions through other ways (e.g., gene expression analysis in barley, cytological observation of infection process) and should also include other cereals to assess the role of both effectors in infection. Basically, we think this question should be part of a separate study rather than an addendum to the current study.

What are the expression profiles of these two HTR genes in different infection and development stages?   Are they specifically expressed in invasive hyphae?

The expression of both genes was induced during infection, but their expression was barely detectable during all four fungal developmental stages, including mycelia, conidia, conidial germination, and appressoria formation. The results are presented in Supplementary Fig. 1.

Do they have NLS? If so, are their NLS necessary for their functions or localization during plant infection?

MoHTR1 contains a putative NLS at residues 140~145 (PGRSKK), which was predicted by WoLF-PSORT. Residues 89~94 (LKPSKG) of MoHTR2 are predicted to be an NLS by NLStradamus. According to Kosugi *et al*. (2009), consensus sequences for known NLSs are: five monopartite NLSs such as Class 1 KR(K/R)R or K(K/R)RK, Class2 (P/R)XXKR(^DE)(K/R), Class 3 KRX(W/F/Y)XXAF, Class 4 (R/P)XXKR(K/R)(^DE), and Class 5 LGKR(K/R)(W/F/Y), and bipartite NLSs $KRX_{10–12}K(KR)(KR)$ or $KRX_{10–12}K(KR)X(K/R)$. Although the predicted NLSs of MoHTR1 and MoHTR2 do not match to any of the known consensus sequences, they appear to be functional in plant cells (Supplementary Fig. 3). It is also possible that MoHTR1 and MoHTR2 contain a yet-to-be-discovered NLS. Follow-up studies are needed to investigate how these proteins move to the host nucleus.

Kosugi, S. *et al*. Six classes of nuclear localization signals specific to different binding grooves of importin alpha. *J. Biol. Chem.* **284**, 478-485 (2009).

Also, the rationale for selecting HTR1 and HTR2 (the sentence on lines 119-120) is not clear (I assume there are more than two HTRs fitting these criteria). Why these two HTR genes? Also, why not just select one?

They were chosen based on the following data. Twenty genes that encode proteins containing both secretion signal and DNA-binding domain were identified from *M. oryzae* genome (Supplementary Table 1). Their expression during the biotrophic stage of infection (Supplementary Fig. 1) was considered. Cellular localization of these proteins during infection, microscopically observed using RFP tagged proteins, was another criterion. Five proteins were associated with the biotrophic interfacial complex (BIC), a structure associated with effector secretion into host cells. Among them, two proteins, MoHTR1 and MoHTR2, were localized in host nuclei during infection. We added these in the main text to help clarify our rationale for selecting HTR1 and HTR2 (lines 125~127).

What is the relationship between Htr1 and Htr2?

MoHTR1 and MoHTR2 have very low sequence similarity (E-value 0.081, coverage 1%). However, they have a C_2H_2 zinc finger domain for DNA binding and a secretion signal at N terminus. Both genes are highly expressed during biotrophic stage of infection.

They differed in protein sequences and DNA binding sites, and affected different genes in rice plants. Why did their overexpression have the same effects on resistance/susceptibility to Mo, Xoo, and Cm? Xoo is an apoplastic bacterial pathogen.

Plants rely on common defense machineries against different pathogens rather than employing pathogen-specific machineries because evolving defense systems customized for individual pathogens is not sustainable. This is why although different pathogens have evolved a wide variety of strategies and effectors to dismantle or suppress host defense, they often target similar genes and processes. Both MoHTR1 and MoHTR2 affected the expression of several immunity-associated genes such as some *PR* genes and phytohormone signaling genes (Supplementary Fig. 5). In addition, 64.5% (240 genes) of the up-regulated genes in *MoHTR1*-OX rice were also up-regulated in *MoHTR2*-OX. Similarly, 36.6% (286 genes) of the down-regulated genes in *MoHTR1*-OX rice were also down-regulated in *MoHTR2*-OX rice (Supplementary Fig. 6). Similar patterns in manipulating rice genes involved in defense by MoHTR1 and MoHTR2 likely caused similar effects on resistance/susceptibility to multiple pathogens.

2. Because the authors used the putative binding sites of Htr1 and Htr2 to identify their target genes in rice, it will be helpful to use additional approaches other than yeast one-hybrid to confirm these binding sites, such as gel mobility shift assays and site-directed mutagenesis of the promoters of selected target genes (OsMYB4, OSsWRK45?).

Thank you for the suggestion. In addition to yeast one-hybrid, we performed a luciferase-based assay by transiently expressing these MoHTR effectors individually in rice protoplasts and confirmed their binding to two target gene promoters *in planta*. In this assay, we cloned the luciferase reporter gene under the promoters of *OsMYB4* and *OsWRKY45* individually and expressed the reporter construct together with each MoHTR effector linked to the TAL activation domain in rice protoplasts. Luminescence increased only when these effectors were expressed along with the reporter. These data strongly support that the MoHTR effectors interact with target gene promoters *in planta*. These additional results are described in lines 212~217 and Fig. 3 d and e.

3. The authors generated Htr1OE and Htr2OE transgenic rice and conducted RNA-seq analysis with these materials. They also showed that both had negative regulation.

What is the percentage of the genes with down-regulated expression levels in Htr1OE or Htr2OE plants have the putative binding sites?

Thank you for asking this and related questions. Among 781 down-regulated genes in *MoHTR1*-OX line, 21 genes (2.69%) have an effector binding element (EBE) in their putative promoter region. Among 798 down-regulated genes in *MoHTR2*-OX line, the putative promoter regions of 75 genes (9.40%) contain EBE. We described the rice genes containing EBE in lines 271~273.

What about the genes up-regulated or not affected have the putative binding sites?

The percentages of EBE-containing up-regulated genes in their promoter are 5.11% (19

genes) and 19.87% (63 genes) in *MoHTR1*-OX and *MoHTR2*-OX, respectively. Some of the not-affected genes contain EBE in their promoter, but the percentages are lower: 3.18% (1,383 genes) and 7.44% (3,237 genes) in *MoHTR1*-OX and *MoHTR2*-OX, respectively. We described this pattern in lines 271~275.

Any enrichment?

As noted above, differentially expressed genes (DEGs) in response to the presence of *MoHTR1* or *MoHTR2* have a higher rate of containing EBE in their promoter compared to those not affected. See lines 271~275.

How many of the genes affected by Htr1OE have the Htr2 recognition site and vice versa?

Among 1,153 DEGs by MoHTR1, 156 genes have the EBE for MoHTR2. Conversely, among 1,115 DEGs by MoHTR2, 39 genes have the EBE for MoHTR1.

The authors generated the htr1 and htr2 deletion mutants in M. oryzae and did some qRT-PCR assays. I am not sure whether they have RNA-seq data of rice leaves infected with these two mutants. If they did, it will be interesting to include (some sentences gave me the impression they had RNA-seq data).

Thank you for this suggestion. We only have qRT-PCR data from Δ*Mohtr*-infected rice, but we hope to pursue such a study in the future.

4. Another question related to the targets of Htr1 and Htr2. How many of these 226 and 650 genes (line 186) were up-regulated or down-regulated?

There are 116 up-regulated and 110 down-regulated genes among 226 genes potentially targeted by MoHTR1. There are 274 up-regulated and 376 down-regulated genes among 650 genes potentially targeted by MoHTR2. We described this pattern in the main text (lines 194~195).

How many of them were common between Htr1OE and Htr2OE plants?

A total of 11 genes were common; five genes were up-regulated, and six genes were down-regulated (see lines 196~197 and Supplementary Table 5).

How many of them or their homologs have been shown to be related to plant immunity?

There are two plant immunity-associated genes among the common target genes of MoHTR1 and MoHTR2. Os09g0572000 is annotated as "pathogenesis-related transcriptional factor" and Os02g0120800 is characterized as *OsRacB*, which has been shown to affect abiotic/biotic stress response. We added this information to Supplementary Table 5.

The authors described three of them on 193-194. Are these three genes the only ones?

Supplementary Tables 3 and 4 list candidate target genes for each MoHTR effector. These genes are predicted to be involved in plant growth, development, and/or abiotic/biotic stress response. Because the expression of *MoHTR1* and *MoHTR2* is highly induced during infection, these candidate genes are regarded as potential plant immunity-related genes. We prioritized to characterize DEGs that are responsive to *M. oryzae* infection and also have

been shown to be involved in defense in previous studies, but other candidates could be also regulated by MoHTR effectors. Characterization of additional target genes via the approaches described in our paper should help expand our understanding of how rice immunity is manipulated by *M. oryza*e.

Are OsMYB4, OsHPL2, and OsWRKY45 induced and/or activated during resistance against bacterial and/or fungal infections (based on published data)? Expression vs activation. Were their expression levels still affected by M. oryzae infection?

Thank you for the questions. It has been reported that the expression of *OsMYB4*, a target of MoHTR1, is induced by cold stress (Vannini *et al*., 2004). Expression of *OsHPL2* and *OsWRKY45*, targets of MoHTR2, was induced by infestation of white-backed planthopper and application of benzothiadiazole, respectively (Gomi *et al*., 2010; Shimono *et al*., 2007). According to Jeon *et al*. (2019), their expression was also increased by *M. oryzae* infection (Supplementary Tables 3 and 4).

Vannini, C. *et al*. Overexpression of the rice *Osmyb4* gene increases chilling and freezing tolerance of *Arabidopsis thaliana* plants. *Plant J*. **37**, 115-127 (2004).
Gomi, K. *et al*. Role of hydroperoxide lyase in white-backed planthopper (*Sogatella furcifera* Horváth)-induced resistance to bacterial blight in rice, *Oryza sativa* L. *Plant J*. 61, 46-57 (2010).
Shimono, M. *et al*. Rice WRKY45 plays a crucial role in benzothiadiazole-inducible blast resistance. *Plant Cell* **19**, 2064-2076 (2007).
Jeon, J. *et al*. Transcriptome profiling of the rice blast fungus *Magnaporthe oryzae* and its host *Oryza sativa* during infection. *Mol. Plant Microbe Interact*. **33**, 141-144 (2019).

Were the expression of OsHPL2 and OsWRKY45 affected in the htr1OE plants? Were the expression of OsMYB4 affected in the htr2OE plants?

None of them were significantly affected. Compared to wild-type rice, expression of *OsHPL2* and *OsWRKY45* did not change in *MoHTR1*-OX rice (1 and 1.04 folds, respectively). Expression of *OsMYB4* is slightly reduced in *MoHTR2*-OX (0.84 fold).

(Just curious, are the Htr1OE and Htr2OE plants normal in growth and development? Many rice genes may have the binding sites of Htr1 and Htr2 and their expression levels were higher than actin)

We could not detect significant difference in growth and development between *MoHTR2*-OX and wild-type plants. However, the growth of *MoHTR1*-OX was reduced to 70% of wild-type rice.

Minor points

1. The title should be more specific. Two nuclear effectors or Nuclear effects Htr1 and Htr2.

We changed the title to “Two nuclear effectors of the rice blast fungus modulate rice immunity via transcriptional reprogramming” to meet the title length limit (15 words).

2. Is Mo necessary to be included in the gene names here? Will HTR1 and HTR2 be more suitable for these two genes?

Conventionally, the *M. oryzae* community uses prefix "Mo" to name newly discovered genes.

3. Line 153, I am not sure whether it has been experimentally proved about moving through plasmodesmata beyond any doubt.

We have not directly proved that they move through plasmodesmata (PD). However, at least three lines of evidence support our conclusion. (1) It has been well documented that plant cytoplasmic proteins can move between cells thorough PD and that the movement is generally dependent on protein size due to PD's size exclusion limit (Zambryski *et al*., 2004); (2) During invasion of rice cells, *M. oryzae* effectors move from initially infected rices to surrounding cells in a protein size dependent manner. The best studied example is the *M. oryzae* effector PWL2. Compared to the frequent cell-to-cell movement observed with PWL2:mRFP (39.3 kD), the larger PWL2:tdTomato (68.3 kD) was rarely observed in adjacent uninvaded rice cells, which is consistent with effector trafficking through PD (Khang *et al*., 2010); (3) In this study, we showed that MoHTR1:mRFP (45.1 kD) and MoHTR2:mRFP (35.6 kD) move cell-to-cell just like PWL2:eGFP:NLS (48.0 kD), which we co-expressed as a control.

Zambryski, P. Cell-to-cell transport of proteins and fluorescent tracers via plasmodesmata during plant development. *J. Cell Biol*. **164**, 165-168 (2004).
Khang, C. H. *et al*. Translocation of *Magnaporthe oryzae* effectors into rice cells and their subsequent cell-to-cell movement. *Plant Cell* **22**, 1388-1403 (2010).

4. What is the average length of 5'UTR for rice?

According to Srivastava *et al*. (2018), it is 259 bp.

Srivastava, A. K., Lu, Y. M., Zinta, G., Lang, Z. B. & Zhu, J. K. UTR-dependent control of gene expression in plants. *Trends Plant Sci*. **23**, 248-259 (2018).

Is 1-kb somewhat too short? (Line 184-186)

We believe that 1-kb for putative promoter length is not too short. According to Joly-Lopez *et al*. (2020), functional elements for rice gene regulation are concentrated within upstream 320 bp from the coding region. Of course, we expect some exceptions, but by expanding the window beyond 1 kb upstream, we run the risk of creating false positives.

Joly-Lopez, Z. *et al*. An inferred fitness consequence map of the rice genome. *Nat. Plants* **6**, 119-130 (2020).

Reviewer #2 (Remarks to the Author):

This study functionally characterized two nuclear effectors of M. oryzae. The authors also generated transgenic rice line expressing these effectors that show increased susceptibility to M. oryzae and Xanthomonas oryzae pv. oryzae, hemibiotrophic pathogens, as well as greater resistance to Cochliobolus miyabeanus, a necrotrophic pathogen. in rice virulence of 192 isolates of Magnaporthe oryzae against several rice varieties carrying known resistance genes. The results and conclusions are appropriate. The manuscript is well written, but lacks

appropriate review of literature. The outcomes should serve as references for fungal geneticist. I support the publication of this manuscript after proper corrections.

I have some comments that the authors should address. There is one problem in the manuscript:
- Lack of the literature review related to the last 6-7 years. There are a couple of excellent papers on fungal nuclear effectors in "high-profile" journals.

Specific comments:

Abstract, Line 28: "no nuclear effectors have been found in fungal pathogens"; The authors should check the literature" There are nuclear effectors described in other fungal pathogens, such as the See1 nuclear effector of the maize smut fungus, Ustilgo maydis (Redkar et al. 2015. Plant Cell. 27: 1332–1351), the CgEP1, a nuclear effector of the maize pathogen Colletotrichum graminicola (Vargas et al., 2016. MPMI. 29:83-95), and the PstGSRE1, of the wheat stripe rust pathogen, Puccinia striiformis f. sp. tritici (Qi et al., 2019. Molecular Plant 12: 1624-1638).

Thank you for pointing out these studies. Although there are some lacks of evidences as nuclear effectors such as nuclear localization during infection or identification of target genes, we added these literatures in the revised manuscript in line 62~66.

Introduction, Line 42: "Plants employ multi-layered systems for protection…" It is a two-layered system. The first layer, also called basal defense, involve PAMP and PRR receptors that triggers PAMP-triggered immunity (PTI). The second layer involves AVR-effectors and NBS-LRR receptors that triggers effector-triggered immunity (ETI).

We appreciate the suggestion and revised it.

Introduction, Line 56-62: the authors should describe in detail the fungal nuclear effectors of U. maydis, C. graminicola and P. striiformis f. sp. Tritici. These effectors were well functionally characterized and there is a rich literature on the topic.

We cited these articles.

Results, Line 195: There many assays to determine DNA-protein interaction, all can give false positives. The most commonly used techniques to determine DNA-protein interactions in vitro techniques are footprinting assays, electrophoretic mobility shift assay, southwestern blotting, yeast one-hybrid assay, phage display and proximity ligation assay. The authors should careful conclude their DNA-protein interaction results. Two accurate in vitro methods should be used.

As noted in comment #2 from reviewer #1, we demonstrated interactions between MoHTR effectors and corresponding target gene promoters *in planta*.

Methods, line 316: Chimeric proteins used in the protein-binding microarray were expressed in E. coli. The authors should use Pichia pastoris or other eukaryotic microorganism for expressing eukaryotic fungal gene for appropriate post translational modifications of protein maturation between other protein modifications processes specific of eukaryotic organisms.

We agree that their expression using Pichia or other eukaryotic expression systems would have been better. However, considering the possibility that effector modification may be species-specific, an alternative expression system based on eukaryotic organism is not a perfect solution. Although we performed a protein-binding microarray using MoHTR effectors expressed in *E. coli*, their binding to the uncovered effector binding elements in eukaryote was successfully demonstrated via yeast one-hybrid experiment (Fig. 3). Of course, we cannot rule out the possibility that these effectors require post-translationally modification for function. This possibility is an interesting avenue to pursue in our future study.

Reviewer #1 (Remarks to the Author):

The authors addressed most of my comments in the revised manuscript or in responses. However, I still have two minor concerns with two points that are parts of my earlier comment #1.

1) The virulence of these two mutants on barley. Most rice blast strains can infection barley. Are the htr1 and htr2 mutants normal in virulence in infection assays with barley seedlings? Do a simple infection assays will not take much time but will determine whether these two nuclear effectors are host-specific or not. Simple infection assays with barley seedlings are sufficient to show whether their virulence on barley is normal or affected. (No need to examine for infectious growth) This suggested experiment is similar to infection assays with Xoo and Cm on HTR1-OE and HTR2-OE transgenic plants the authors did.

2) The authors responded to my comment on 'What is the relationship between HTR1 and HTR2? They differed in protein sequences and DNA binding sites, and affected different genes in rice plants. Why did their overexpression have the same effects on resistance/susceptibility to Mo, Xoo, and Cm? Xoo is an apoplastic bacterial pathogen. The responses from the authors are not so convincing to me and I think it is better to address this point in Discussion. HTR1 and HTR2 share little sequence similarity and only 11 DEGs (5 up- and 6 down-regulated). How can these HTR1-OE and HTR2-OE had similar effects on resistance/susceptibility to Mo, Xoo, and Cm? (totally different pathogens) Related discussion may be of interest to readers.

Reviewer #2 (Remarks to the Author):

Kim et al., 2020. Nature Communications

General comments:

The revised manuscript has been improved significantly. All major comments have been addressed, and most suggested modifications have been included into this new version of the manuscript.

This remarkable study showed the importance of the nuclear effectors of M. oryzae for host cell reprogramming. I highly recommend to publish this manuscript in its present form.

Reviewer #1 (Remarks to the Author):

The authors addressed most of my comments in the revised manuscript or in responses. However, I still have two minor concerns with two points that are parts of my earlier comment #1.

1) The virulence of these two mutants on barley. Most rice blast strains can infection barley. Are the htr1 and htr2 mutants normal in virulence in infection assays with barley seedlings? Do a simple infection assays will not take much time but will determine whether these two nuclear effectors are host-specific or not. Simple infection assays with barley seedlings are sufficient to show whether their virulence on barley is normal or affected. (No need to examine for infectious growth) This suggested experiment is similar to infection assays with Xoo and Cm on HTR1-OE and HTR2-OE transgenic plants the authors did.

We infected barley with the two mutants and KJ201. Virulence of both mutants was reduced, suggesting that the function of these two effectors is not rice specific. This result is shown in Supplementary Fig. 8 and described in Results (lines 279~281). We also slightly modified Discussion (lines 362~367) to highlight this new result.

2) The authors responded to my comment on 'What is the relationship between HTR1 and HTR2? They differed in protein sequences and DNA binding sites, and affected different genes in rice plants. Why did their overexpression have the same effects on resistance/susceptibility to Mo, Xoo, and Cm? Xoo is an apoplastic bacterial pathogen. The responses from the authors are not so convincing to me and I think it is better to address this point in Discussion. HTR1 and HTR2 share little sequence similarity and only 11 DEGs (5 up- and 6 down-regulated). How can these HTR1-OE and HTR2-OE had similar effects on resistance/susceptibility to Mo, Xoo, and Cm? (totally different pathogens). Related discussion may be of interest to readers.

Further studies are needed to understand how the overexpression of these two nuclear effectors significantly affected rice defense against disparate types of pathogens. However, we can certainly speculate a likely mechanism underpinning this effect. We modified Discussion to address this recommendation (see lines 372~385). Thanks for reminding us of the potential significance of this question and the need to highlight it better.

Reviewer #2 (Remarks to the Author):

The revised manuscript has been improved significantly. All major comments have been addressed, and most suggested modifications have been included into this new version of the manuscript.

This remarkable study showed the importance of the nuclear effectors of M. oryzae for host cell reprogramming. I highly recommend to publish this manuscript in its present form.

We very much appreciate many suggestions you have provided.

Reviewer #1 (Remarks to the Author):

The authors have properly address all my comments. Thanks
